# Triterpene Derivatives as Potential Inhibitors of the RBD Spike Protein from SARS-CoV-2: An In Silico Approach

**DOI:** 10.3390/molecules28052333

**Published:** 2023-03-02

**Authors:** Mayra Avelar, Laura Pedraza-González, Adalgisa Sinicropi, Virginia Flores-Morales

**Affiliations:** 1 Laboratorio de Síntesis Asimétrica y Bio-Quimioinformática (LSAyB), Ingeniería Química (UACQ), Universidad Autónoma de Zacatecas, Campus XXI Km 6 Carr. Zac-Gdl, Zacatecas 98160, Mexico; 2 Department of Chemistry and Industrial Chemistry, University of Pisa, Via Moruzzi 13, 56124 Pisa, Italy; 3 Department of Biotechnology, Chemistry and Pharmacy, University of Siena, 53100 Siena, Italy; 4 Institute of Chemistry of Organometallic Compounds (CNR-ICCOM), Via Madonna del Piano 10, 50019 Sesto Fiorentino, Italy; 5 CSGI, Consorzio per lo Sviluppo dei Sistemi a Grande Interfase, 50019 Sesto Fiorentino, Italy

**Keywords:** triterpenes, SARS-CoV-2, spike protein, RBD, molecular docking, molecular dynamics

## Abstract

The appearance of a new coronavirus, SARS-CoV-2, in 2019 kicked off an international public health emergency. Although rapid progress in vaccination has reduced the number of deaths, the development of alternative treatments to overcome the disease is still necessary. It is known that the infection begins with the interaction of the spike glycoprotein (at the virus surface) and the angiotensin-converting enzyme 2 cell receptor (ACE2). Therefore, a straightforward solution for promoting virus inhibition seems to be the search for molecules capable of abolishing such attachment. In this work, we tested 18 triterpene derivatives as potential inhibitors of SARS-CoV-2 against the receptor-binding domain (RBD) of the spike protein by means of molecular docking and molecular dynamics simulations, modeling the RBD S1 subunit from the X-ray structure of the RBD-ACE2 complex (PDB ID: 6M0J). Molecular docking revealed that at least three triterpene derivatives of each type (i.e., oleanolic, moronic and ursolic) present similar interaction energies as the reference molecule, i.e., glycyrrhizic acid. Molecular dynamics suggest that two compounds from oleanolic and ursolic acid, **OA5** and **UA2**, can induce conformational changes capable of disrupting the RBD-ACE2 interaction. Finally, physicochemical and pharmacokinetic properties simulations revealed favorable biological activity as antivirals.

## 1. Introduction

The emergence of a novel coronavirus, SARS-CoV-2, was identified in Wuhan, China, in December 2019. Since then, around 6.7 million of human deaths worldwide have been associated with the disease caused by SARS-CoV-2, namely COVID-19, according to the World Health Organization (WHO; January 2023, https://covid19.who.int/). Although vaccination is now a reality, its effect depends on physical characteristics of vaccinated individuals. For instance, it is known that vulnerable groups could exhibit a weak response to vaccination and still be prone to serious complications [1,2,3,4,5]. Therefore, the development of alternative treatments for COVID-19 is still necessary. In this regard, in recent years, there has been a growing interest in carrying out in silico studies for designing new drugs or repurposing the existing ones. In fact, for the specific case of drug design strategies for identifying potential anti-SARS-CoV-2 inhibitors, computational approaches have proven advantageous not only in speeding up the time to study large sets of potential candidates but also in allowing rational design through an understanding of the action mechanism of virus inhibition [6,7,8,9,10,11,12,13,14,15,16,17,18,19,20,21,22,23,24]. Although most of the reported methodologies consist of the combination of a screening step with molecular docking and a refining step with molecular dynamic (MD) simulations, there is current growing interest in the development of automated high-throughput machine learning (ML) approaches [13,16].

To date, several proteins have been proposed as a target for drug design against SARS-CoV-2. Among them, spike protein (S protein) is considered one of the principal objectives because it mediates the entrance of coronavirus into the host cell by cell recognition and membrane fusion. The S protein is composed of two functional subunits, namely the S1 subunit, which includes the receptor-binding domain (RBD), and the S2 subunit, which is also called the membrane fusion domain [25]. Considering that the RBD mediates the first step of infection by the recognition of angiotensin-converting enzyme 2 (ACE2) in human cells, a promising alternative for COVID-19 treatment is the search for small molecules capable of blocking the RBD-ACE2 interaction [12,21,22,24,26]. In this regard, key amino acid residues mediating such interactions, hereinafter referred to as a “hot spot”, have been resolved by structural analysis [27,28,29,30].

More specifically, both in vitro experiments and in silico studies have revealed hot spot amino acid residues that intermediate the RBD-ACE2 specific recognition site [27,31,32,33]. For instance, Xu et al. found, by site directed mutagenesis, two important sites in the RBD domain for specific ACE2 recognition. They report that a T470–T478 loop mutation showed a complete loss to the ACE2 binding. Moreover, a single-mutation Y505A also abolishes the interaction [27]. On the other hand, Maffucci et al. found two hot spot binding sites by molecular dynamics and in silico alanine scanning corresponding to BS1—L455, F456, F486, N487, Y489 and Q493—and BS2—Y449, Q498, T500, N501 and Y505 [32]. According to these studies, there is a specific region that contributes to the binding affinity of RBD-ACE2 and could be used for in silico drug design by the search of small molecules interacting in this region. Although the first attempt is to block the protein–protein interactions with the presence of small peptides or other molecules, a promising alternative is to find allosteric modulators that could induce conformational changes in the RBD capable of breaking the protein–protein interaction [12,34,35]. These molecular modulators could come from plants as secondary metabolites (SM) or their semi-synthetic derivatives, specifically those that exhibit antiviral properties. Indeed, it is a matter of fact that natural compounds present potential anti-SARS-CoV-2 properties [12,24,36,37,38,39,40].

Triterpenoids, widely used in traditional herbal medicine, represent an interesting case of natural compounds playing an important role in plant defense. The antiviral activity of these molecules against human immunodeficiency virus 1 (HIV-1), hepatitis B virus (HBV), hepatitis C virus (HCV), influenza A virus (IAV), Ebola virus (EBOV) and SARS-CoV has been reviewed [41,42,43,44,45,46]. Interestingly, it has been demonstrated that it is the potential anti-SARS-CoV-2 activity of triterpenoid molecules that makes them a target for drug development against COVID-19 disease [47,48]. Remarkably, the inhibitory potential of glycyrrhizic acid (GA) and licorice-saponin against S protein has been identified by means of in silico and in vitro experiments [47,49]. Additionally, Li et al. found that triterpenoid derivatives with the 3-Ob-chacotriosyl oleanolic acid skeleton were potent inhibitors of the S2 subunit from the spike protein, blocking the membrane fusion [50]. Furthermore, the Food and Drug Administration (FDA) has approved the emergency use of vaccines against COVID-19, and the use of antiviral drugs, i.e., remdesivir, for adults and some pediatric patients, as well as the immune modulators baricitinib and tocilizumab (1 February 2023, https://www.fda.gov). However, there are no in vitro experiments that support these approved drugs as inhibitors of the S1 subunit of the S protein of SARS-CoV-2.

In this work, we evaluate whether 18 triterpene derivatives from oleanolic (OA), moronic (MA) and ursolic (UA) acids present either similar or enhanced-potential antiviral activity against SARS-CoV-2 compared with the reference GA. To this aim, we combined molecular docking and molecular dynamics simulations to study the effect of the triterpene derivatives against the RBD domain of the S1 subunit at the S protein. The latter was modeled from the X-ray structure of the SARS-CoV-2 RBD–ACE2 complex (PDB ID: 6M0J [51]) elucidated by Lan et al. Molecular docking suggests that at least one derivative of each triterpenic acid presents docking scores in the range of the reference molecule GA, which suggests the probable inhibition effect of these compounds against the S protein. Moreover, molecular dynamics simulations revealed that OA and UA derivatives can induce conformational changes at the interaction site similar to GA, indicating its potential effect for blocking the RBD-ACE2 interaction.

## 2. Results and Discussion

As mentioned above, several studies revealed that the RBD subunit S1 of the SARS-CoV-2 S protein mediates the first step of COVID-19 infection by the recognition of the angiotensin-converting enzyme 2 (ACE2) in human cells. In this work, we explore the potential antiviral activity of 18 triterpene derivatives, hereinafter referred as ligands, from oleanolic (OA), moronic (MA) and ursolic (UA) acids by evaluating their possible role in blocking the RBD-ACE2 interaction through the formation of a more stable RBD–ligand complex. The 18 ligands, listed and illustrated in Table 1 were selected from previous reports of antidiabetic studies [52,53,54] searching for a new use of these molecules against COVID-19, and triterpenoids were reported as good antivirals. As observed, OA, MA and UA were included as a control, with GA as the reference molecule, for a total of 22 compounds. The GA molecule was chosen for reference since its structure corresponds to a triterpenoid derivative and has been proven, by in vitro experiments, as an inhibitor of the RBD-ACE2 interactions.

### 2.1. Molecular Docking Modeling

In order to explore the possible binding modes and identify low-energy binding poses that lead to the formation of a complex between the RBD and the target ligands, molecular docking calculations for each of the 22 aforementioned molecules against the RBD subunit S1 of SARS-CoV-2 spike protein were performed. As an attempt to mimic the structural conditions determined experimentally to favor the RBD-ACE2 interaction (i.e., considering each target ligand rather than the ACE2 receptor), the geometry of the RBD used for docking calculations was extracted from the crystallographic structure of the RBD-ACE2 complex [51] (see Section 3.2). Since a semiflexible docking was performed, during the simulations the geometry of the RBD was fixed to the crystallographic coordinates, while the ligands were able to be movable and flexible. This setup is widely employed, allowing the ligand to adapt to conformational changes during the interaction with the receptor [12]. Moreover, two hot spots of the RBD S1 subunit proposed by Maffucci et al. [32], namely BS1 (L455, F456, F486, N487, Y489 and Q493) and BS2 (Y449, Q498, T500, N501 and Y505), were selected as binding sites.

After protein and ligand preparation (see Section 3.1 and Section 3.2), each of the 22 ligands were docked into both the BS1 and BS2 hot spots by using the procedure described in Section 3.3. We remark that in molecular docking calculations, the selection of the box size parameter is crucial for obtaining meaningful results in terms of docked pose prediction. While a narrow search space might not provide all the probable poses, a large-size docking box might promote the formation of unrealistic conformations. In our simulations, we followed the strategy reported by Feinstein and Brylinski [55], namely that by using an optimized dimension of the search space 2.9 times larger than the radius of gyration (Rg), the accuracy of the docked compound improves, enhancing the compound ranking (see Equation (1), Section 3.3).

**Figure 1:**
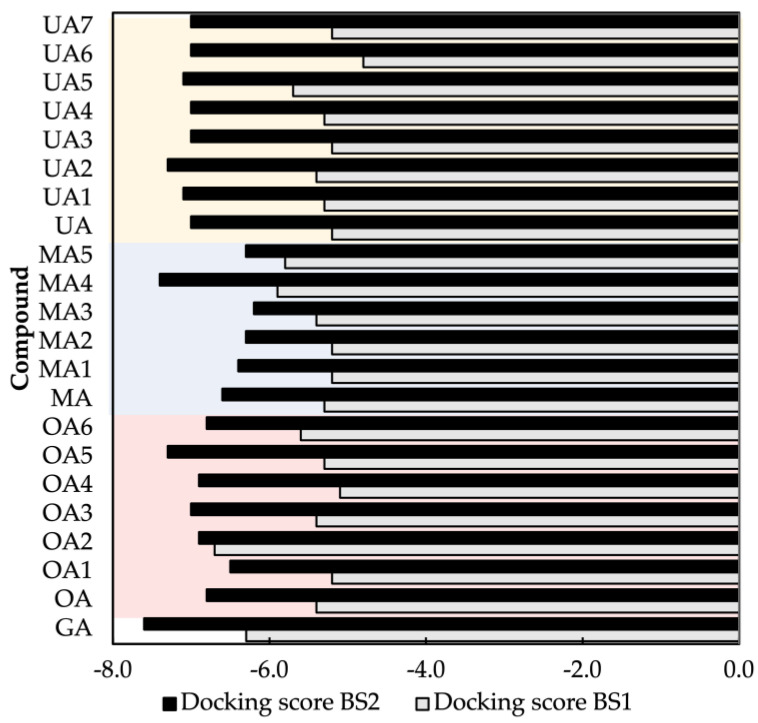
Autodock Vina docking scores for BS1 and BS2 sites for all the studied compounds. Docking scores are given in kcal/mol. Yellow, blue and red color boxes represent UA, MA and OA sets, respectively.

Table 1 and Figure 1 report the docking scores, which correspond to the lowest-energy binding pose computed for each of the 22 (i.e., 18 target triterpene derivatives, **OA**, **MA**, **UA** and **GA**) compounds docked with either the BS1 or BS2 hot spots. The corresponding interacting residues of the RBD with the target ligand using a cutoff of 5 Å are also shown. These results provide complementary information on two main points: (i) the potential efficacy of the target ligands in blocking the RBD-ACE2 complex formation by the production of a stable RBD–ligand interaction, and (ii) the most favorable hot spot mediating such an interaction. The first point is rationalized by means of comparison with a reference molecule, i.e., glycyrrhizic acid (GA), that has been experimentally demonstrated to exhibit an antiviral activity against SARS-CoV-2 by blocking the RBD-ACE2 interaction [47]. The second point is achieved by comparing the docking binding scores of each target ligand, including GA, with respect to both BS1 and BS2, selecting the hot spot producing a lower binding energy.

We first analyze the docking scores of all the ligands in terms of the most favorable hot spot for hosting the respective RBD–ligand interaction. At a first glance, as observed in Figure 1, it is possible to notice that in all the cases, the docking scores of BS2 (i.e., −6.2 to −7.6 kcal/mol) are lower (more negative) than the ones of BS1 (i.e., −4.8 to −6.7 kcal/mol). This holds true for the case of the reference molecule GA, with docking scores of −6.2 and −7.6 kcal/mol for BS1 and BS2, respectively. This can be explained according to the number of hydroxyl groups presented in the molecule that could form H-bonds with the protein. Moreover, the results found in all the compounds suggest that BS2 is the hot spot that contributes to a more favorable RBD–ligand energy interaction, with the RBD-GA complex the most favorable one. We remark that in the selected docking poses GA is the only compound interacting with the five amino acids composing BS2, while none of the other 21 compounds interact with Y449 (see Table 1). In the following, we will focus our analysis exclusively on the binding site BS2 of the RBD.

We now analyze the BS2 docking scores of the 18 target compounds and compare them with the values of the control molecules, i.e., UA −7.0 kcal/mol, OA −6.8 kcal/mol and MA −6.6 kcal/mol. As reported in Table 1, the set of UA derivatives present the overall highest docking scores (−7.0 to −7.3 kcal/mol), followed by OA (−6.5 to −7.3 kcal/mol) and finally MA (−6.2 to −7.4 kcal/mol). It is worth noting that in both OA and UA sets, most of the six derivatives present scores equal to or higher than the respective control compound, except for **OA1** (−6.5 kcal/mol). In contrast, in the case of MA set, the compound **MA4** (−7.4 kcal/mol) is the only one with an enhanced score. In light of these observations, the most straightforward strategy could be to focus on the six ligands of the UA set for further investigations on RBD-UA-based interactions. However, as discussed below, we decided to study a single derivative representative of each set by selecting the one with the docking binding score similar to GA (−7.6 kcal/mol) and analyze in detail their similarities/differences with respect to the latter in terms of the stability and structure of their RBD–ligand complex. Accordingly, we selected **OA5** (−7.3 kcal/mol), **MA4** (−7.4 kcal/mol) and **UA2** (−7.3 kcal/mol).

**Figure 2:**
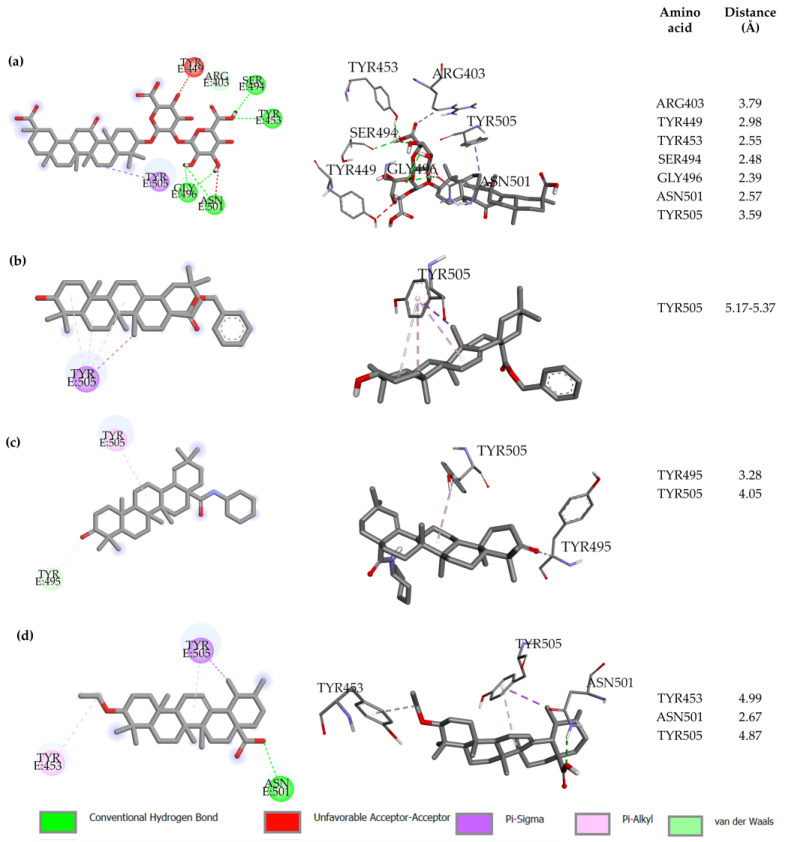
2D (left) and 3D (right) interaction diagram for: (**a**) GA reference molecule, (**b**) **OA5**, (**c**) **MA4** and (**d**) **UA2** derivatives. Residue–ligand distances are shown. All diagrams were constructed with Discovery studio 2021. Interaction-type color labels are displayed.

We now turn to comparing the structural features of the RBD(BS2)-**X** complex, with **X** = **OA5**, **MA4** and **UA2**, with the structural features of the RBD(BS2)-GA complex, in order to gain a further understanding of the interactions that lead to the top-ranked docking scores among all the studied compounds. In this regard, we analyze the GA-BS2, **OA5**-BS2, **MA4**-BS2 and **UA2**-BS2 docked structures, as well as the type of molecular interactions. As illustrated in Figure 2, for each case, we generated an interaction diagram that shows the amino acid residues of RBD interacting with the ligand in the most favorable (lowest-energy) binding pose. For further information about the contact residues in the BS2 site for all derivatives, see Figure S1.

When focusing on the reference compound GA, an interesting observation is that it interacts not only with all the five residues composing the binding site BS2 (i.e., Y449, Q498, T500, N501, Y505) but also with seven other residues of the RBD (none of them belonging to BS1) (see Table 1). Moreover, the most relevant molecular interactions between RBD and GA when docking BS2 can be summarized as follows (see Figure 2a). GA presents four H-bond interactions with Y453, S494, G496 and N501 at 2.6, 2.5, 2.4 and 2.6 Å, respectively (measured between the donor and the acceptor atoms). In addition, it displays Pi–sigma interactions with Y505 at 3.6 Å distance (Figure 2a). Although GA displays four hydrogen bonds that contribute to favor the total energy interaction, it presents two unfavorable interactions, the first one between the oxygen of the hydroxyl group in the aromatic ring of Y449, with the second between the oxygen of one hydroxyl group in the GA structure. Both oxygen atoms are placed 2.9 Å away from each other, generating repulsive interactions. On the other hand, the oxygen atom from the carbonyl group of the backbone in the N501 amino acid residue presents repulsive interactions with a hydroxyl group of GA, with both oxygen atoms at a 3 Å distance (Figure 2a, red lines). These repulsive interactions are unfavored the energy interaction of the complex; however, due to the attractive interactions, they are still the most favorable among all the studied compounds.

A similar analysis of the top three selected compounds revealed the following: the **OA5** derivative showed one Pi–sigma at 3.7 Å and three Pi–alkyl interactions at 5 Å, with an average distance between the aromatic ring of Y505 and the oleanolic skeleton of **OA5** (see Figure 2b). Additionally, **MA4** only presents one Pi–alkyl interaction with Y505 at 4.7 Å (Figure 2c). Finally, **UA2**, displays one hydrogen bond with N501 at 2.7 Å, as well as Pi–alkyl and Pi–sigma interactions with Y505 (4.9 Å and 3.9 Å, respectively), and one Pi–alkyl with Y453 at 5.0 Å (Figure 2d). Although the interactions of the three top compounds with the RBD S1 subunit seem to be less complex than in the case of GA, one interesting finding is that all of them present interactions (either Pi–sigma or Pi–alkyl) with the residue Y505. Remarkably, in vitro experiments that comprise single-point mutations [27] have identified Y505 as a hot spot amino acid in the interaction between RBD and ACE2, and it has been suggested that such a residue plays a key role in blocking the interaction among proteins, inhibiting virus replication.

**Figure 3:**
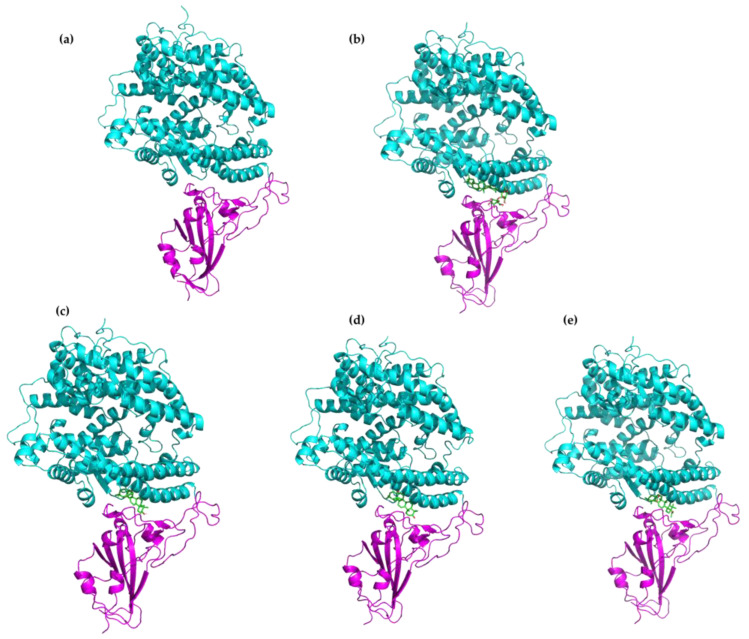
Protein–protein docking poses of the top selected compounds: (**a**) RBD-ACE2 complex, (**b**) RBD-ACE2 GA complex, (**c**) RBD-ACE2 **OA5** complex, (**d**) RBD-ACE2 **MA4** complex, (**e**) RBD-ACE2 **UA2** complex. The RBD subunit is highlighted in magenta color, the ACE2 in cyan color and the ligand is shown in ball-and-stick representation in green color.

On the other hand, as an attempt to evaluate whether the inclusion of the top selected ligands influences the stability of the RBD-ACE2 complex, we have computed the binding affinity between RBD and ACE2 before and after the formation of the RBD(BS2)-**X** interactions. To this aim, we performed protein–protein (i.e., RBD-ACE2) docking calculations based on the knowledge-based iterative scoring function ITScorePP, employing the HDOCK server [56,57]. Afterwards, the binding free energy for the docked poses was estimated with the so-called molecular mechanics energies combined with the generalized Born and surface area continuum solvation (MM/GBSA) method, implemented in the HawDock Server [58,59,60,61]. Interestingly, we found that in all the cases the presence of the ligands at the BS2 site of the RBD and those located in the interface with the ACE2 (Figure 3) unfavored the interaction energy of the RBD-ACE2 complex (less negative value). More specifically, the MM/GBSA-based binding free energy of the RBD-ACE2 complex in the absence of any ligand is −86.73 kcal/mol, and this value increases to −73.66, −73.89, −65.26 and −72.80 kcal/mol for complexes with GA, **OA5**, **MA4** and **UA2**, respectively. These binding energy values give us an estimation about the effect of the ligand in protein–protein interactions, suggesting that the complex connections at the BS2 site could be disrupted by the presence of the selected top ligands.

Summarizing, a molecular docking screening of 22 ligands allowed us to select BS2 as the more favorable binding site of the RBD S1 subunit to induce an interaction with triterpene acid derivatives. Furthermore, although many docking poses with favorable energetic interactions between the studied ligands and BS2 were identified for the 18 target compounds in the RBD-ACE2 interface, we selected the three top derivatives (i.e., **OA5**, **MA4** and **UA2**) and the reference compound GA for further analysis. As shown below, in order to study in detail the stability and dynamical behavior of the predicted docked RBD(BS2)–ligand complexes, we carried out molecular dynamics calculations. We investigate whether or not MD confirms our hypothesis that Y505 plays a key role in the formation of the RBD(BS2)–ligand complexes. Future work will be devoted to expanding the number of candidates for MD simulations.

### 2.2. Molecular Dynamics Simulations

Molecular dynamics (MD) simulations are crucial to evaluate the protein–ligand stability of the RBD(BS2)-**X** complexes predicted with molecular docking (see Section 2.1). Unlike the semiflexible approach used in docking (see above), MD allows to model the protein flexibility in a fully hydrated environment. Therefore, starting from the GA-BS2, **OA5**-BS2, **MA4**-BS2 and **UA2**-BS2 docked structures, we performed two independent MD 100 ns replicas following the procedure described in Section 3.4. In parallel, to measure the induced conformational changes in the protein structure due to the presence of the ligand, the unbonded RBD domain was also modeled.

We first evaluated the structural stability of the RBD protein by means of the root-mean-square deviation (RMSD) measure, illustrated in Figure S2 of the Supporting Information (SI). As shown in this figure, all modeled systems reach equilibrium at the simulated time (100 ns). The average backbone RMSD for unbound RBD, **OA5**-RBD and GA-RBD complexes was found to be 2.7 Å, whilst the **MA4**-RBD complex was 2.4 Å and **UA2**-RBD was 2.6 Å, with maximum movements around 4.0 Å in all compounds. Although the GA molecule induces higher movements in the protein, it remains stable during the last 40 ns of simulation (Figure S2, SI). Moreover, as reported in Table S1, the secondary structure of the RBD was not perturbed by the presence of the ligands.

**Figure 4:**
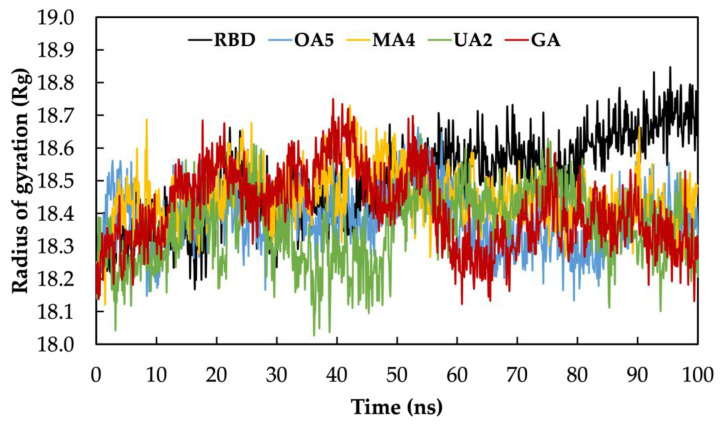
Radius of gyration computed along the MD simulation time.

In addition to measuring the conformational stability of the RBD(BS2)-**X** complexes, we calculated the radius of gyration (Rg). This property gives us an idea of the compactness of the protein structure and the folding stability during MD simulation. We found that, for all the complexes, the Rg is lower than the RBD subunit in the last 40 ns of simulation (Figure 4), indicating a greater compactness of the system. Additionally, the values in all the complexes remained relatively constant, hence the complex is considered to be stably folded, and the overall protein structure is stable after ligand binding [26].

**Figure 5:**
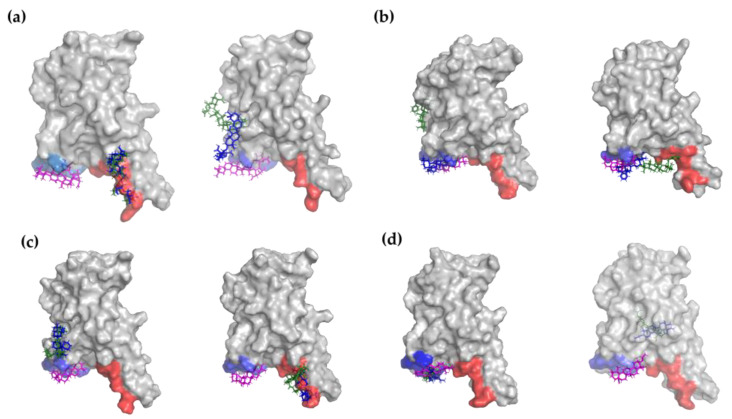
Ligand positions at 0 ns (magenta), 50 ns (blue) and 100 ns (green) of first (left) and second (right) replica of: (**a**) GA reference molecule, (**b**) **OA5**, (**c**) **MA4** and (**d**) **UA2** derivatives. All diagrams were constructed with PyMOL. BS1 site is colored in red and BS2 in blue.

**Figure 6:**
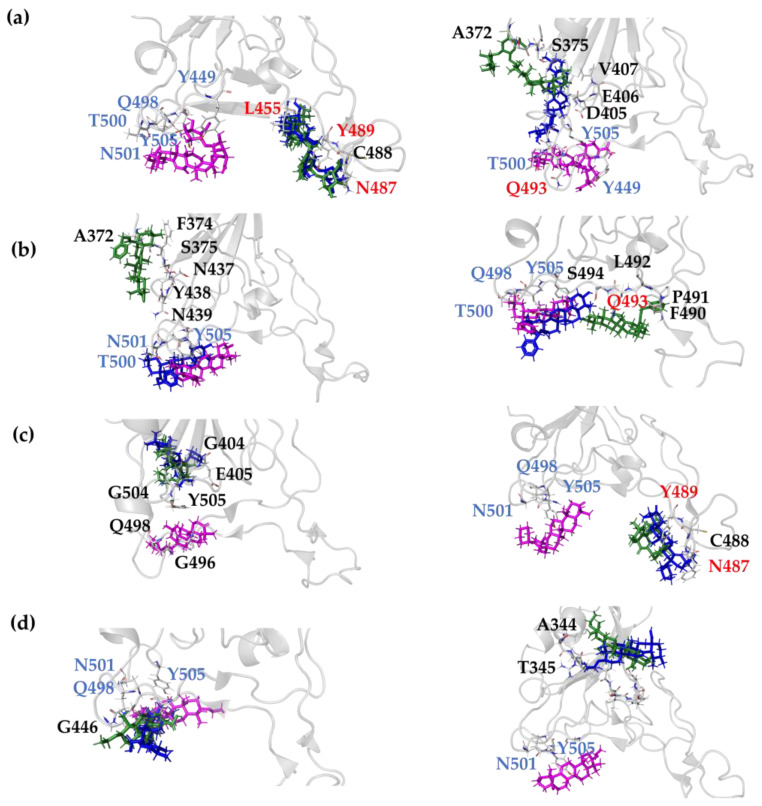
Trajectory snapshots at 0 ns (magenta), 50 ns (blue) and 100 ns (green) of first (left) and second (right) replica of: (**a**) GA reference molecule, (**b**) **OA5**, (**c**) **MA4** and (**d**) **UA2** derivatives. All diagrams were constructed with PyMOL. Grey ribbons represent RBD domain while interacting residues are displayed as sticks and labeled; red labels represent the amino acids at BS1 and blue labels the amino acids at BS2.

Once we ensured that the system reached equilibrium, an analysis of the stability of the interactions predicted by molecular docking calculations was performed. We found that all the studied compounds maintained interactions with the protein in both replicas; however, as illustrated in Figure 5, some molecules lose contact with the amino acids from the RBD-ACE2 interface, i.e., the first attempt to block protein–protein interactions. In this regard, an estimation of the new contacts must be interpreted in order to establish if the protein interaction inhibition could take place due to conformational changes in the RBD subunit by an allosteric site. In the case of GA, we observed a different interaction pattern in the two replicas. In the first replica, GA unbinds the docked position (Figure 6a, left, magenta representation) after around 15 ns of simulation; interestingly, in the remaining simulation time, it was positioned at the BS1 site (Figure 6a, left, blue and green representation), interacting with the residues of L455 and N487-Y489. In the second replica, GA also abandoned the initial position after 24 ns of simulation; however, it reaches another position near the BS2 site with contact amino acids: A372-S375, G404-G407 and G502-Y505 (Figure 6a, right, blue and green representation). One interesting finding is that, even though this molecule did not last at the docked position, GA remains near the two hot spot regions, which could contribute to preventing the interactions between RBD and ACE2.

Remarkably, the **OA5** derivative was found to be the compound that prevails most of the time in the docked region. In the first replica, **OA5** stayed at the BS2 site until 65 ns of simulation (Figure 6b, left, magenta and blue representation); afterwards, it moved to the region composed of the residues N437-N439 and A372-S375, which is not far from the BS2 region (Figure 6b, left, green representation). Notice that GA also reached a position in close contact with residues A372-S375. Moreover, in the second replica, the compound keeps its position under the 100 ns simulation, moving only at the end of the MD at a region between BS1 and BS2 (F490-S494) but always at the interface of RBD-ACE2 (Figure 6b, right). Hence, MD simulations support our hypothesis that **OA5** could be a potential molecule to inhibit protein–protein interactions and avoid SARS-CoV-2 infection.

On the other hand, **MA4** is the molecule whose interactions with the amino acids at the interface of RBD-ACE2 last for less time, with less than the 80% of the time (200 ns) at this region. In the first replica, it goes to the region near the residues G404-E406 and G504-Y505 (Figure 6c, left, blue and green representation), whilst in the second replica, it remains at the BS1 F486-Y489 (Figure 6c, right, blue and green representation). Finally, **UA2** in the first replica does not leave the docked position at BS2 (Figure 6d, left), whilst in the second replica, it moves away at the 7 ns simulation, interacting with A344-A352 residues at 16 Å from the BS2 (Figure 6d, right, blue and green representation). These results indicate that, although BS2 seems to be the most favorable hot spot, BS1 could play an important role in the RBD-ACE2 interaction, as predicted by Maffucci et al. [32].

**Figure 7:**
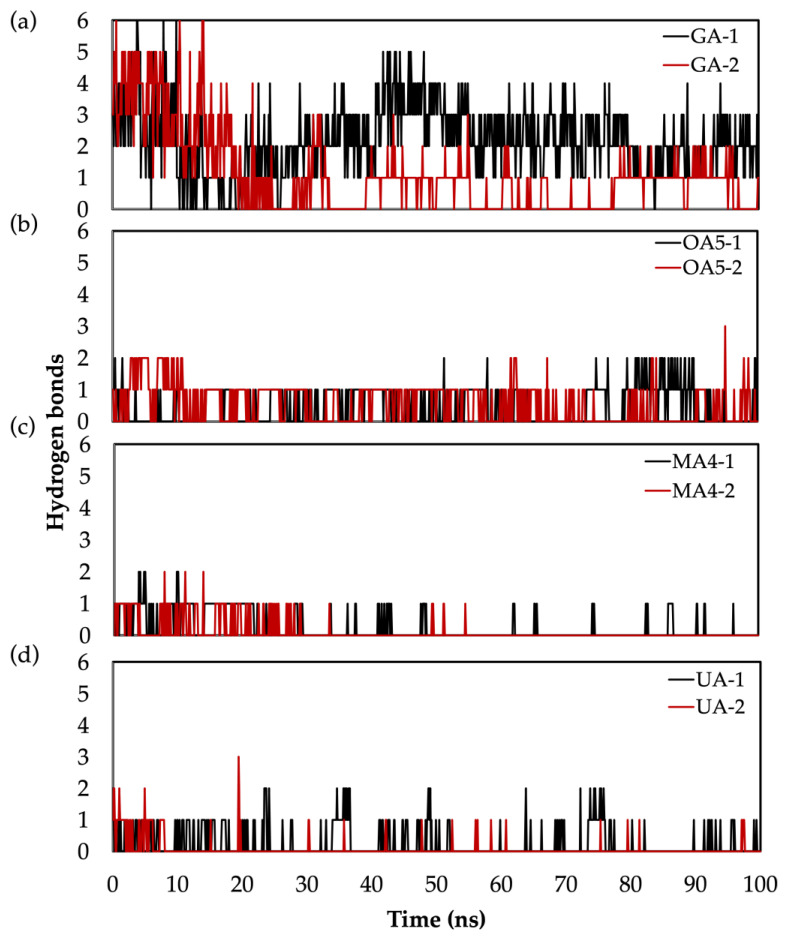
Number of H-bonds during MD for: (**a**) GA, (**b**) **OA5**, (**c**) **MA4** and (**d**) **UA2**. Black representation corresponds to replica 1, and red to replica 2.

Furthermore, among all the ligand interactions with the protein, the existence of hydrogen bonds (H-bond) is crucial for the stability of the protein–ligand complex. Therefore, the analysis of the H-bond during the MD simulation reveals that GA presents a major number of this kind of interaction, with an average of three and two H-bonds for the first and second replicas, respectively (see Figure 7a). On the other hand, **MA4** shows a lower number of H-bonds, being completely lost in the second replica after 50 ns of simulation (Figure 7c). **OA5** presented the most stable H-bond number in both replicas with at least 1 H-bond, and **UA2** also had stable interactions in the first replica; however, in the second replica after 10 ns simulation, only occasional H-bonds are present. From these results, we can conclude that GA and **OA5** form the most stable complexes among the four systems, with **MA4** forming the least stable.

**Figure 8:**
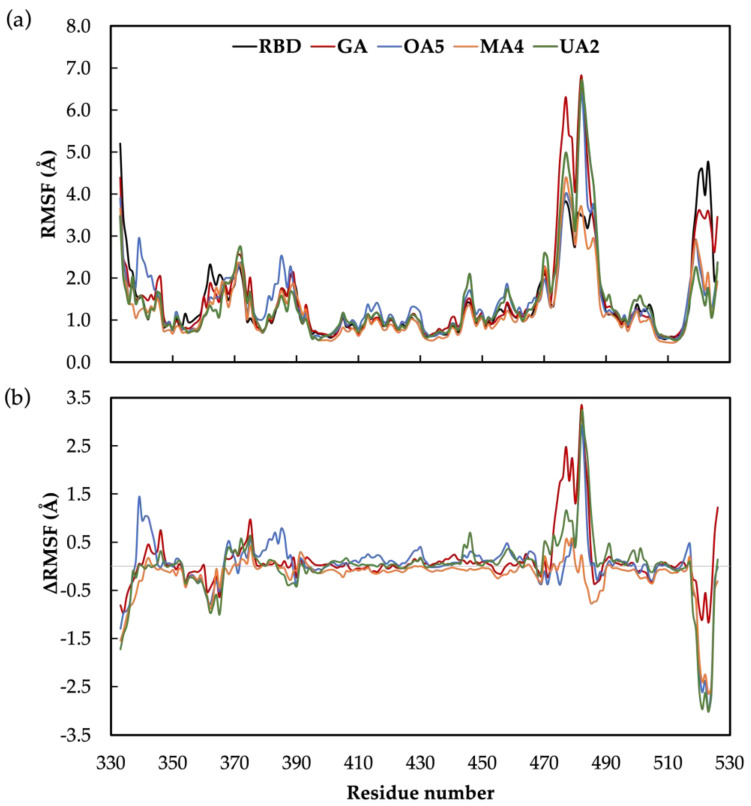
(**a**) RMSF values for the unbonded RDB domain and all the studied complexes of the protein–ligand; (**b**) ΔRMSF values relative to unbonded RBD domain of RMSF. Values are given in Å.

Afterwards, to have a deeper understanding of the potential influence of these molecules in RBD-ACE2 interactions by altering the protein conformation of the RBD, we analyze the effect on the protein flexibility by means of the root-mean-square fluctuation (RMSF). The obtained values are displayed in Figure 8a and correspond to an average of the two replicas; the individual RMSF is presented in Figure S3 of the SI. For all protein–ligand complexes and the unbonded RBD domain, a higher-flexibility region was found at the region composed of amino acids A475-Y487 (values are presented in Table S2 of the SI). These results are in agreement with the work of Williams et al., who also found great flexibility in such a region corresponding to the E471-P491 loop within the RBD domain [62].

In order to compare the effect of the ligand, the ΔRMSF relative to the unbonded RBD was calculated and presented in Figure 8b; positive values correspond to an increase in the protein flexibility whilst negative values represent a decrease in the protein flexibility. We found that GA, **OA5** and **UA2** induce higher fluctuations in the region composed of the N481-E484 amino acids. Likewise, Alvarado et al. found deviations in the N481-V483 amino acids due to the interaction of the RBD with the luteolin molecule. They proposed that this higher oscillation affects protein–protein interactions between RBD and ACE2, consequently inhibiting virus infection. Related to this, we found for the reference molecule (GA) a maximum increase of 3.3 Å at the G482, with 2.2, 2.4 and 1.6 Å for N481, V483 and E484, respectively (see Figure 7). GA remains for almost all the simulated time at the interface of RBD-ACE2, but not in the region reported as crucial for protein–protein interaction [27]; nonetheless, it induces a conformational change near the residues T470-T478 that could provoke the loss of protein–protein interactions and therefore the inhibition of the virus infection.

Indeed, as already reported, GA has been demonstrated to avoid SARS-CoV-2 infection by in vitro experiments [47]. Moreover, Li et al. demonstrated, by surface plasmon resonance measurements, that GA is capable of preventing the contact between RBD and ACE2 by interacting with a recombinant S protein. In this work, among the studied compounds, **OA5** and **UA2** showed comparable RMSF deviations to GA at the N481-E484 region (Figure 8 and Table S2). In this regard, the **OA5** compound presents a 2.9 Å deviation at G482, and **UA2** presents an ΔRMSF of 3.2 Å in this position. On the other hand, **MA4** does not show a difference in the protein flexibility at this region, giving an ΔRMSF of 0.2 Å considering a cutoff of 0.3 Å for a ligand-induced fluctuation, as reported previously [22,63].

Related to the interaction region (Figure 5 and Figure 6) and the ΔRMSF (Figure 8), the two replicas of GA show different RMSF values, Figure S3b, with the second replica showing larger movements than the first replica. Since the first replica presented lower flexibility at the T470-P490 region, as shown in Figure 6a, because of the interaction of GA in this case near the BS1 site, the ligand interaction appears not to induce an effect on mobility. On the other hand, the second replica shows the highest amounts of fluctuation and interactions in the region, including A372-S375 and G404-V407. Furthermore, the **OA5** derivative shows the same fluctuations (Figure S3c) in both replicas presenting interactions at the interface of RBD-ACE2 (F490-Y505) and the residues of A372-S375 out of the interface. These results from GA and **OA5** suggest that the site at the A372-S375 residues is an allosteric modulator site that contributes to the flexibility at the region A475-N487 and could disrupt protein–protein interactions [34]. Additionally, **MA4** displays diverse effects in each replica (Figure S3d). The first one shows higher movements at the region S477-P479 and interactions near the BS2 site (G404-E406, V503-P507), while the second replica does not show differences with the unbonded RBD when interacting in the F486-Y489 region. Finally, the **UA2** derivative shows the highest influence on the flexibility when it interacts at the BS2 site (replica 1, Figure S3e), although it presents fewer fluctuations when the contact occurs at the residues A344-S349 (replica 2, Figure S3e).

**Figure 9:**
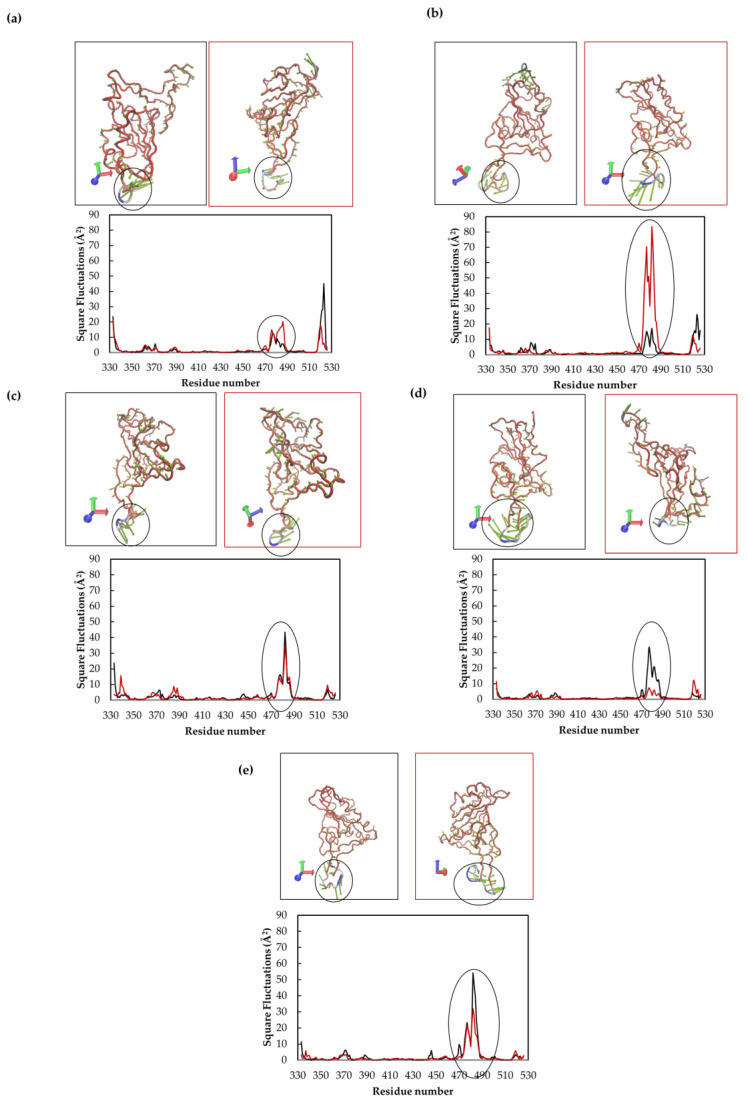
PCA mode 1 and square fluctuations, performed with ProDy application, for: (**a**) RBD, (**b**) GA reference molecule, (**c**) **OA5**, (**d**) **MA4** and (**e**) **UA2** derivatives. All diagrams were constructed using NMWiz plugin in VMD. T475-P487 is marked by a circle. Black representation corresponds to replica 1, and red corresponds to replica 2.

In order to confirm the observed motion in the T475-P487 region, principal component analysis (PCA) was used to study the flexible region by the essential motions of the protein. It is known that PCA is a dimensionality reduction technique widely used for analyzing the motion of complicated systems with many degrees of freedom [64], allowing the efficient representation of each point in the MD trajectory as a point in an essential plane [65]. As shown in Figure 9, our analysis indicates that the key motions of the RBD(BS2)-**X** complexes were examined. PCA revealed that the first mode explains the flexibility in the T475-P487 loop, showing a highly dynamic component in this region. For instance, the second replica of GA presented higher square fluctuations, related to the T475-P487 loop shown in Figure 9b (black circle), than the first one.

Summarizing, we found two hot spots for the potential inhibition of RBD-ACE2 interactions by triterpenoid derivatives. The first region corresponds to the BS2 site, proposed by Maffucci et al., located at the RBD-ACE2 interface, whose connection could be blocked by ligand interactions with the RBD amino acids. Moreover, the presence of the **OA5** molecule in this site provoked higher flexibility at the N481-E484 region (Figure 3). This increase in flexibility was also observed when the compounds interact with the A372-S375 amino acids, the latter representing a potential allosteric modulator site. Alvarado et al. also reported a distal binding region, composed of the residues Y369, F377 and K378, inducing a conformational change in the residues N481-V483 and provoking a lower number of contacts between the S protein and the ACE2 receptor, disrupting RBD-ACE2 interactions. In this regard, triterpenoids that induce conformational changes in this region could act as potential S protein inhibitors.

### 2.3. Physychochemical and Pharmacokinetic Properties

In silico predictions of the physicochemical and the pharmacokinetic properties of potential molecules as therapeutic structures could save time and money when performing evaluations in preclinical trials. The comparison with the parameters proposed by Muegge, Veber, Ghose and Egan are made to complement the values of properties that are not found in the rule of five, and because, currently, not all commercial drugs exactly resemble the values proposed by Lipinski; therefore, when there is a discrepancy with Lipinski values, there is the possibility of justifying use through other empirical rules.

The calculations of the physicochemical and the pharmacokinetic properties were performed for the top three selected compounds (**OA5**, **MA4** and **UA2**), the GA reference molecule, and two additional references: (i) remdesivir (RS), an approved FDA antiviral drug which acts as an RNA polymerase inhibitor, and (ii) umifenovir (arbidol (UM)), an indole derivative which has proven anti-influenza activity but is not an FDA-approved drug in western countries; however, it has shown favorable results against COVID-19 [66] and was proposed as a potential inhibitor of the S1 subunit of the spike protein by in silico studies [67].

**Figure 10:**
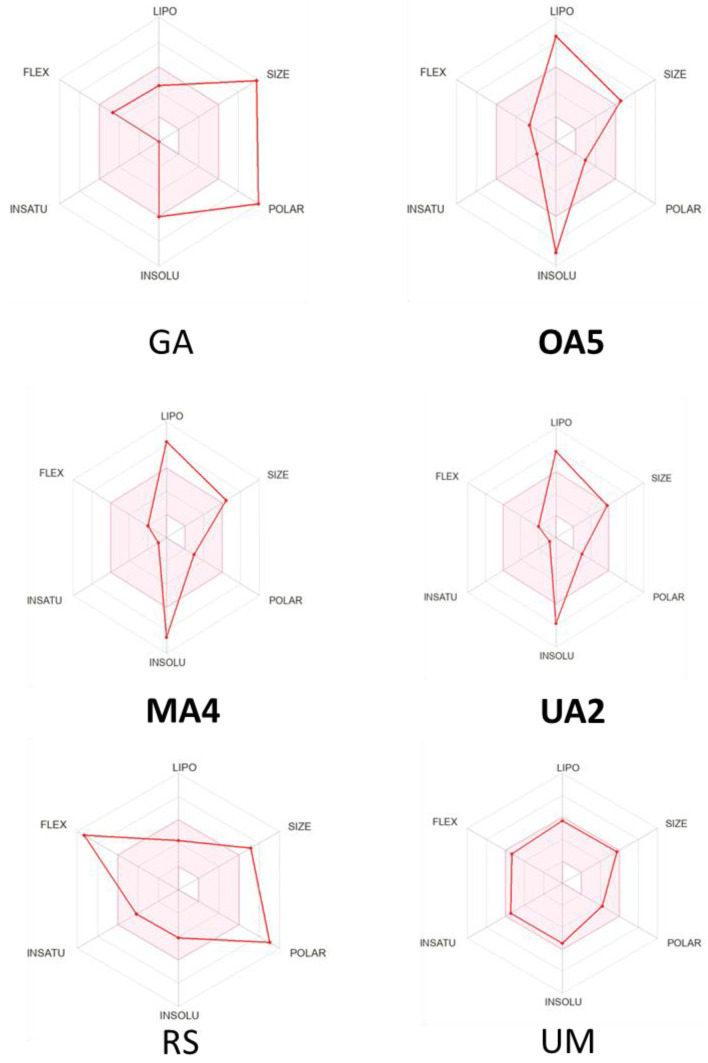
Bioavailability radars for GA, RS, UM, **OA5**, **MA4** and **UA2**. The pink area represents the optimal range for each property.

As shown in Figure 10, the bioavailability radars of the selected compounds **OA5**, **MA4** and **UA2**, as well as the reference molecule GA, are out of range in at least two physicochemical properties, such as lipophilicity and insolubility. According to these results, and also considering the Egan diagram (Figure S4, SI), the reference molecules and the triterpene derivatives present low bioavailability for oral absorption, except for UM. As shown in Table 2 and Table 3, ADME properties for GA, RS, **OA5** and **MA4** derivatives exceed the limits of the molecular weight. Moreover, the reference molecules GA and RS presented a TPSA higher than 140 Å^2^, indicating a low cell membrane permeability, whilst the selected derivatives showed TPSA values suitable for cell permeability. According to the LogP_o/w_ values, all the studied compounds (except UM) exhibited no gastrointestinal absorption with respect to Egan (WLOGP) and Lipinski (MLOGP). Moreover, GA reference and the selected derivatives (**OA5**, **MA4** and **UA2**) presented poor solubility due to their LogS values (ESOL). The low bioavailability and LogP_o/w_ for oral adsorption shown by the studied derivatives GA and RS are based on the rule of five, considering only passive diffusion as a transport mechanism. However, some of these structures may use the ATP-dependent mechanism, which would facilitate their passage through the membrane or even suggest a different route of administration than an oral one. Physicochemical and pharmacokinetic properties for all the studied compounds are found in Tables S3 and S4 of the SI.

In order to evaluate the possible biological activities, focusing particularly on antiviral activities, such as influenza and 3CLpro inhibitors, bioactivity parameters were calculated. Considering that the range of probability for favorable activity is found with values greater than 0.69, discrete values greater than or equal to 0.40, and non-favorable values below 0.30 [68], we calculated the bioactivity by using the PASS online server. As shown in Table 4, the compounds GA, **OA5**, **MA4** and **UA2** were the ones that showed the best results as antivirals, particularly as anti-influenza, with favorable probability values of activity (Pa of 0.737–0.833), being better than RS and UM. Additionally, related to the probability of acting as an inhibitor of 3CLPro from SARS-CoV2, the **OA5** derivative displayed a discrete probability of activity, while **UA2** presented an unfavorable probability. It is important to highlight that RS and UM did not report this biological activity on the used platform.

## 3. Materials and Methods

### 3.1. Triterpene Derivatives DFT Calculations

Geometry optimization and harmonic frequency calculations of all ligand compounds (SMILES at Table S5, SI) were performed at the DFT level of theory [69,70,71], employing the functional hybrid B3LYP [72,73] and 6 − 31 + G(d,p) basis sets using the Gaussian 16 quantum chemistry package (G16) [74]. Optimized minima were verified with the number of imaginary frequencies, determined as zero (NImag = 0) in all cases. All calculations were carried out in gas phase.

### 3.2. Protein Model Preparation

The structure of RBD subunit from SARS-CoV-2 was extracted from the crystallographic structure PDB ID 6M0J [51]. The RBD subunit was prepared using the Protein Preparation Wizard tool in Maestro software (release 2019.2) to add hydrogen atoms and delete crystallographic water, counterions (Zn^2+^ and Cl^−^) and NAG ligands. Standard protonation states were assigned for all the ionizable residues by setting a pH of 7.4 using PROPKA [75,76]. After macromolecular preparation, the molecular charge was +2.

### 3.3. Molecular Docking Calculations

Molecular docking calculations were carried out using a proposed protocol that relies on the use of the open-source program AutoDock Vina [77]. Such a protocol can be described as follows. First, optimized ligand and RBD xyz structures were converted to pdbqt files using MGL tools version 1.5.6. The search space was set around two hot spots reported by Maffucci et al., namely BS1 with coordinates x: −38.621, y: 39.731, z: 1.564 and corresponding to amino acids L455, F456, F486, N487, Y489 and Q493, and BS2 centered at x: −36.355, y: 20.471, z: 2.322, related to residues Y449, Q498, T500, N501 and Y505. The interaction box size was fit according to the ligand size, as reported by [55], using Equation (1) to establish xyz cubic dimension.

Box size (x,y,z) = 2.857 × Rg,(1)

where Rg is the radius of gyration of the docking compound, computed with the measure rgyr analysis tool of VMD [78], which uses the Equation (2).

(2) rgry2=(∑i=1nω(i)(r(i)−r¯)2)(∑i=1nω(i))

where r(i) is the position of the ith atom and $\bar{r}$ is the weighted center.

Exhaustiveness was set to 10 in all calculations with an energy range of 3.0 kcal/mol. In total, *N* = 10 replicas were carried out for each ligand, with a maximum of 20 number modes. The *N* = 10 replicas were produced from 10 independently generated random seeds to guarantee an exhaustive search of the conformational space. Best pose according to the function score for each triterpene derivative docked with RBD subunit was selected for further molecular dynamics simulations analysis.

The above-described protocol was implemented in a command-line Python3-based driver that automates the whole procedure, requiring as only input the files of the optimized structure for RBD and target ligand. The docking score values of the *N* = 10 replicas of each top selected compound are reported in Table S6 of the SI.

Protein–protein docking was performed using the prepared model (see Section 3.2) with the ACE2 chain as receptor and the RBD as ligand. Calculation was carried out with the HDock server [56,57]. Using the docked poses, the binding free energy was estimated based on molecular mechanics energies combined with the generalized Born and surface area continuum solvation (MM/GBSA) method, implemented in the HawDOCK Server [58,59,60,61].

### 3.4. Molecular Dynamics Simulation

MD simulations were performed using the Desmond package from Schrödinger suite 2019-2 [79]. Periodic boundary conditions were set using a cubic box of 15 Å from the protein surface, with a 7.4 working pH and 0.15 M ion strength. Explicit solvent molecules were treated with the TIP3P water model. Two replicas of 100 ns were carried out for each ligand complex using the OPLS_2005 force field [80]. Prior production run, a minimization–relaxation protocol was carried out, composed by five steps: (a) 100 ps Brownian dynamics NVT simulation with solute heavy atoms restrained at 10 K; (b) 12 ps NVT simulation with heavy atoms restrained at 10 K; (c) 12 ps NPT with restrains on solute heavy atoms at 10 K; (d) 12 ps NPT simulation at 300 K and solute heavy atoms restrained; (e) 24 ps NPT simulation with no atoms restrained at 300 K. For steps (b–e), the Berendsen algorithm for thermostat/barostat was employed [81]. Production run was performed at 310 K and 1 bar using the NPT ensemble with the Berendsen thermostat and barostat to maintain temperature and pressure, applying 1 ps and 2 ps relaxation time, respectively. The RESPA integrator was used with 2 fs time step, and short-range interactions were modeled with a 12 Å cutoff.

### 3.5. In Silico Prediction of Physicochemical and Pharmacokinetic Properties of Triterpene Acid Derivatives

Cheminformatics and bioactivity prediction data (including ADMET, pharmacokinetics and medicinal chemistry properties) were calculated using canonical SMILES (Simplified Molecular Input Line Entry Specification) sequences retrieved from the pdb files of the DFT optimized structures (Section 3.1 using Maestro software (release 2019.2). The chemical space analysis was focused on four physicochemical properties (PCP) of pharmaceutical relevance, molecular weight (MW), TPSA (Topological Polar Surface Area), cLogP (octanol/water partition coefficient) and solubility (ESOL) predicted with the SwissADME web tool (http://www.swissadme.ch/index.php; accessed date: 10 November 2022). Bioavailability radars and Lipinski’s [82], Ghose’s [83], Veber’s [84], Egan [85] and Muegge’s [86] rules were also calculated using SwissADME. Additionally, the toxicological profile and the antiviral activity against influenza and 3-chymotripsin-like protease inhibitor were computed using the Prediction of Activity for Substance (PASS) online software (http://www.way2drug.com/passonline/; accessed date: 10 November 2022).

### 3.6. Data Analysis

Two-dimensional interaction diagrams for molecular docking analysis were analyzed with the Ligand Interaction Diagram tool in Maestro (Schrödinger 2019.2 release). Simulation Interaction Diagram tool from Desmond (Schrödinger 2019.2 release) was used to compute the root-mean-square deviation (RMSD) and the root-mean-square fluctuation (RMSF) of the trajectories. Principal component analysis (PCA) was performed using the Desmond trajectories and converted to dcd format using the ProDy open-source package [87,88,89], and the Normal Mode Wizard (NMWiz) plugin in VMD was used for visual comparative analysis [87,88].

## 4. Conclusions

Eighteen triterpene derivatives from oleanolic, ursolic and moronic acids were studied against SARS-CoV-2 inhibition by molecular docking and molecular dynamics simulations. The docking scores of 10 out of 18 studied compounds showed similar values of around −7.0 kcal/mol as the reference molecule (glycyrrhizic acid, −7.6 kcal/mol) in the most favorable interaction site (i.e., BS2) formed by Y449, Q498, T500, N501 and Y505. The top selected compounds displayed interaction energies of −7.3, −7.4 and −7.3 kcal/mol for **OA5**, **MA4** and **UA2** derivatives, respectively. Since the GA molecule has been reported to block the RBD-ACE2 interaction by in vitro and in silico experiments, the selected triterpenoids could also have a potential inhibition effect. Furthermore, molecular dynamics simulations showed that the reference molecule, GA, induced a conformational change at residues N481-E484 that could disrupt protein–protein interactions. **OA5** and **UA2** derivatives presented comparable movements with the reference molecule at this site and could act as potential inhibitors through this mechanism. The change in the flexibility of the RBD S1 subunit was related to the interaction of these three compounds (GA, **OA5** and **UA2**) with the region composed of the A372-S375 amino acids, which could act as an allosteric modulator of movement in the N481-E484 site, disrupting the RBD-ACE2 interactions and consequently inhibiting virus replication. According to their physicochemical and pharmacokinetic predicted properties, the **OA5**, **MA4** and **UA2** derivatives and GA show a low oral bioavailability and low gastrointestinal adsorption. Nevertheless, all the selected compounds presented favorable potential as antivirals. The results found in this work open the possibility to perform further in vitro experiments to validate the anti-SARS-CoV-2 activity of **OA5**, **MA4** and **UA2** derivatives for use as COVID-19 therapeutics.

## Figures and Tables

**Table 1:** Docking scores, interacting residues (5 Å cutoff) and chemical structure of the studied compounds and the reference compound (GA). Docking scores are reported in kcal/mol.

Compound Key Name	Chemical Structure	BS1 Docking Score	BS2 Docking Score	Residues at a 5 Å Sphere of Interaction
GA	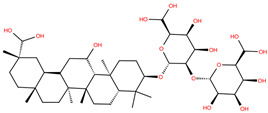	−6.3	−7.6	**BS1**: K417, Y453, L455, F456, E484, G485, F486, N487, C488, Y489, F490, L492, Q493 **BS2**: R403, Y449, Y453, S494, Y495, G496, F497, Q498, T500, N501, G502, Y505
OA	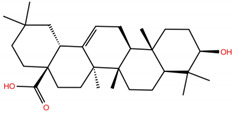	−5.4	−6.8	**BS1**: K17, L455, F456, E484, G485, F486, N487, Y489 **BS2**: R403, Y453, Q493, S494, Y495, G496, Q498, T500, N501, G502, Y505
**OA1**	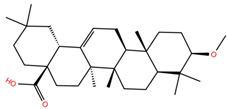	−5.2	−6.5	**BS1**: K417, L455, F456, E484, G485, F486, N487 Y489 **BS2**: R403, Y453, S494, Y495, G496, Q498, T500, N501, G502, Y505
**OA2**	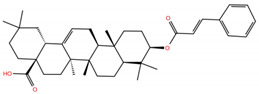	−6.7	−6.9	**BS1**: L455, F456, Y473, A475, E484, N487, Y489, F490, Q493 **BS2**: R403, E406, K417, Y453, S494, Y495, G496, F497, Q498, T500, N501, Y505
**OA3**	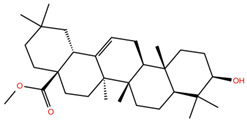	−5.4	−7.0	**BS1**: K417, L455, F456, E484, G485, F486, N487, C488, Y489, F490 **BS2**: R403, Y453, S494, Y495, G496, Q498, T500, R501, G502, Y505
**OA4**	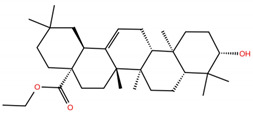	−5.1	−6.9	**BS1**: K417, L455, F456, E484, G485, F486, N487, C488, Y489, F490 **BS2**: R403, Y453, S494, Y495, G496, Q498, T500, N501, G502, Y505
**OA5**	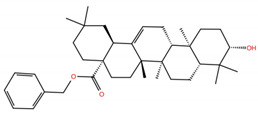	−5.3	−7.3	**BS1**: K417, L455, F456, E484, N487, Y489, F490, L492, Q493 **BS2**: G446, Y453, S494, Y495, G496, Q498, T500, N501, G502, Y505
**OA6**	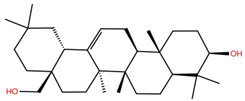	−5.6	−6.8	**BS1**: K417, L455, F456, E484, G485, F486, N487, C488, Y489 **BS2**: R403, Y453, Y495, G496, Q498, T500, N501, G502, Y505
MA	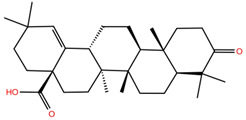	−5.3	−6.6	**BS1**: K417, L455, F456, E484, G485, C488, Y489, F490, Q493 **BS2**: R403, Y453, S494, Y495, G496, Q498, T500, N501, G502, Y505
**MA1**	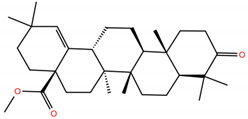	−5.2	−6.4	**BS1**: K417, L455, F456, E484, G485, C488, Y489, F490, Q493 **BS2**: R403, Y453, S494, Y495, G496, Q498, T500, N501, G502, Y505
**MA2**	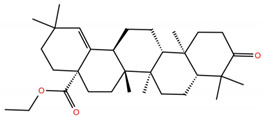	−5.2	−6.3	**BS1**: K417, L455, F456, Y473, A475, E484, N487, Y489 **BS2**: R403, Y453, S494, Y495, G496, Q498, T500, N501, G502, Y505
**MA3**	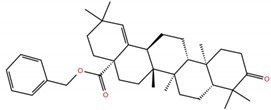	−5.4	−6.2	**BS1**: L455, F456, E484, G485, F486, C488, Y489, Q493 **BS2**: R403, Y453, S494, Y495, G496, Q498, T500, N501, G502, Y505
**MA4**	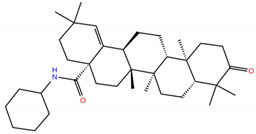	−5.9	−7.4	**BS1**: K417, L455, F456, A475, E484, N487, Y489, Q493 **BS2**: R403, Y453, S494, Y495, G496, Q498, T500, N501, G502, Y505
**MA5**	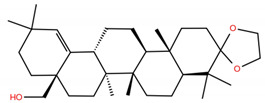	−5.8	−6.3	**BS1**: K417, L455, F456, E484, Y489, F490, Q493 **BS2**: R403, Y449, Y453, S494, Y495, G496, Q498, T500, N501, G502, Y505
UA	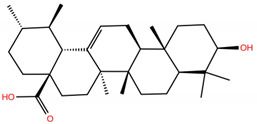	−5.2	−7.0	**BS1**: K417, L455, F456, E484, G485, C488, Y489, F490, Q493 **BS2**: R403, Y453, S494, Y495, G496, Q498, T500, N501, G502, Y505
**UA1**	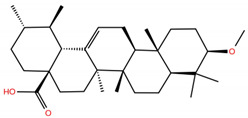	−5.3	−7.1	**BS1**: K417, L455, F456, E484, G485, C488, Y489, F490, Q493 **BS2**: R403, E406, Y453, S494, Y495, G496, Q498, T500, N501, G502, Y505
**UA2**	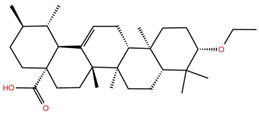	−5.4	−7.3	**BS1**: K417, Y453, L455, F456, E484, G485, F486, C488, Y489, F490, Q493 **BS2**: R403, E406, Y453, S494, Y495, G496, Q498, T500, N501, G502, Y505
**UA3**	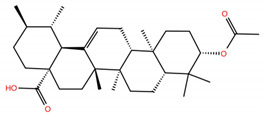	−5.2	−7.0	**BS1**: K417, L455, F456, E484, G485, F486, C488, Y489, F490, Q493 **BS2**: R403, E406, Y453, S494, Y495, G496, Q498, T500, N501, G502, Y505
**UA4**	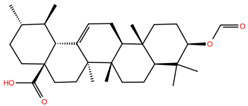	−5.3	−7.0	**BS1**: K417, Y453, L455, F456, E484, G485, F486, C488, Y489, F490, Q493 **BS2**: R403, E406, Y453, S494, Y495, G496, Q498, T500, N501, G502, Y505
**UA5**	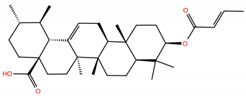	−5.7	−7.1	**BS1**: K417, Y421, L455, F456, R457, Y473, A475, E484, Y489, Q493 **BS2**: R403, E406, Y453, S494, Y495, G496, Q498, T500, N501, G502, Y505
**UA6**	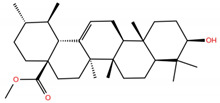	−4.8	−7.0	**BS1**: L455, F456, E484, G485, C488, Y489, Q493 **BS2**: R403, E406, Y453, S494, Y495, G496, Q498, T500, N501, G502, Y505
**UA7**	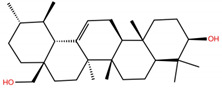	−5.2	−7.0	**BS1**: K417, L455, F456, E484, C488, Y489, F490, Q493 **BS2**: R403, E406, Y453, S494, Y495, G496, Q498, T500, N501, G502, Y505

**Table 2:** Structural and physicochemical properties of the top selected derivatives and the reference molecules.

Compound	M. Wt g/mol	TPSA Å^2^	Log P _o/w_		LogS (ESOL)	HBA	HBD	Rotatable Bonds	Druglikeness *					Bioavailability Score
			WLOGP	MLOGP					Lipinski	Ghose	Veber	Egan	Mugue	
**OA5**	546.02	46.53	8.74	6.83	−8.94	3	1	4	2	4	0	1	1	0.17
**MA4**	535.84	46.17	7.52	5.90	−8.62	2	1	3	2	4	0	1	1	0.17
**UA2**	484.75	46.53	8.13	6.20	−7.64	3	1	3	1	4	0	1	1	0.85
GA	833.01	279.68	−0.20	−0.67	−6.05	16	12	7	3	3	1	1	4	0.17
RS	602.58	213.36	2.21	0.18	−4.12	12	4	14	2	3	2	1	3	0.17
UM	477.41	80.00	4.87	3.59	−5.45	4	1	8	0	0	0	0	0	0.55

* Number of rule violations. Red numbers indicate out of range values.

**Table 3:** Pharmacokinetic and medicinal chemistry properties.

Compound	Pharmacokinetics								Medicinal Chemistry		
	GI Absorption	BBB Permeant	P-gp Substrate	CY1A2 Inhibitor	CYP2C19 Inhibitor	CYP2C9 Inhibitor	CYP2D6 Inhibitor	CYP3A4 Inhibitor	PAINS	Brenk	Leadlikeness
**OA5**	Low	No	No	No	No	No	No	No	0	1	2
**MA4**	Low	No	No	No	No	No	No	No	0	1	2
**UA2**	Low	No	No	No	No	No	No	No	0	1	2
GA	Low	No	Yes	No	No	No	No	No	0	2	1
RS	Low	No	Yes	No	No	No	No	Yes	0	1	2
UM	High	No	No	No	Yes	Yes	Yes	Yes	1	0	3

**Table 4:** Biological properties of the top selected derivatives and the reference molecule. Values predicted with PASS online server.

Compound	Antiviral (Influenza)		3CLpro (Human Coronavirus) Inhibitor	
	Pa	Pi	Pa	Pi
**OA5**	0.764	0.004	0.361	0.005
**MA4**	0.746	0.004	NR	NR
**UA2**	0.737	0.004	0.278	0.041
GA	0.833	0.002	NR	NR
RS	0.216	0.174	NR	NR
UM	0.740	0.004	NR	NR

Pa: Probability of activity. Pi: Probability of inactivity. NR: Not reported.

## Data Availability

Not applicable.

## Supplementary Materials

The following supporting information can be downloaded at: https://www.mdpi.com/article/10.3390/molecules28052333/s1, Figure S1: 2D interaction diagram for all triterpene derivatives. All diagrams were constructed with Discovery studio 2021. Interaction-type color labels are displayed; Figure S2: RMSD protein backbone values: (a) RBD domain, (b) GA-RBD complex, (c) **OA5**-RBD complex, (d) **MA4**-RBD complex, (E) **UA2**-RBD complex. Black representation corresponds to replica 1, and red corresponds to replica 2; Figure S3: RMSF CA values: (a) RBD domain, (b) GA-RBD complex, (c) OA5-RBD complex, (d) MA4-RBD complex, (e) UA2-RBD complex. Black representation corresponds to replica 1, red corresponds to replica 2; Figure S4. Egan’s boiled egg diagram for all the studied compounds and the reference molecule. Neither molecule is a substrate of the P-gp; Table S1: secondary structure percentage from the final frame in the 100 ns molecular dynamics simulation; Table S2: RMSF values, in Å, for the region T470-F490 for both replicas; Table S3: structural and physicochemical properties for all the derivatives and the reference molecule. Table S4: pharmacokinetic and medicinal chemistry properties for all the studied compounds; Table S5: SMILES of the studied compounds and the reference compound (GA); Table S6: Molecular docking scores of the top selected compounds with *N* = 10 from 10 independently generated random seeds. The most favorable energy binding is shown for each seed.

## Sample Availability

Samples of the compounds are not available from the authors.
