# Supplementary material for: Triterpene Derivatives as Potential Inhibitors of the RBD Spike Protein from SARS-CoV-2: An In Silico Approach
Source: Molecules. 2023 Mar 2;28(5):2333. doi: 10.3390/molecules28052333 (PMC10005606; doi:10.3390/molecules28052333)
Supplement: Supplementary file 1 [file molecules-28-02333-s001.zip › molecules-2219958-supplementary.pdf]

Supporting Information:

Triterpene derivatives as potential inhibitors of the RBD spike protein from SARS-CoV-2: an *in silico* approach

Mayra Avelar [1*], Laura Pedraza-González [2], Adalgisa Sinicropi [3,4,5] and Virginia Flores-Morales [1*]

[1] Laboratorio de Síntesis Asimétrica y Bio-quimioinformática (LSAyB), Ingeniería Química (UACQ), Universidad Autónoma de Zacatecas, Campus XXI Km 6 Carr. Zac-Gdl, Zacatecas 98160, Zacatecas, Mexico; (MA) mayra.avelar@uaz.edu.mx; VF-M (virginia.flores@uaz.edu.mx)

[2] Department of Chemistry and Industrial Chemistry, University of Pisa, Via Moruzzi 13, 56124, Pisa, Italy; laura.pedraza@dcci.unipi.it

[3] Department of Biotechnology, Chemistry and Pharmacy, University of Siena, 53100 Siena, Italy; adalgisa.sinicropi@unisi.it

[4] Institute of Chemistry of Organometallic Compounds (CNR-ICCOM), Via Madonna del Piano 10, 50019 Sesto Fiorentino, Italy.

[5] CSGI, Consorzio per lo Sviluppo dei Sistemi a Grande Interfase, 50019 Sesto Fiorentino, Italy.

*Correspondence: MA, mayra.avelar@uaz.edu.mx; VF-M, virginia.flores@uaz.edu.mx

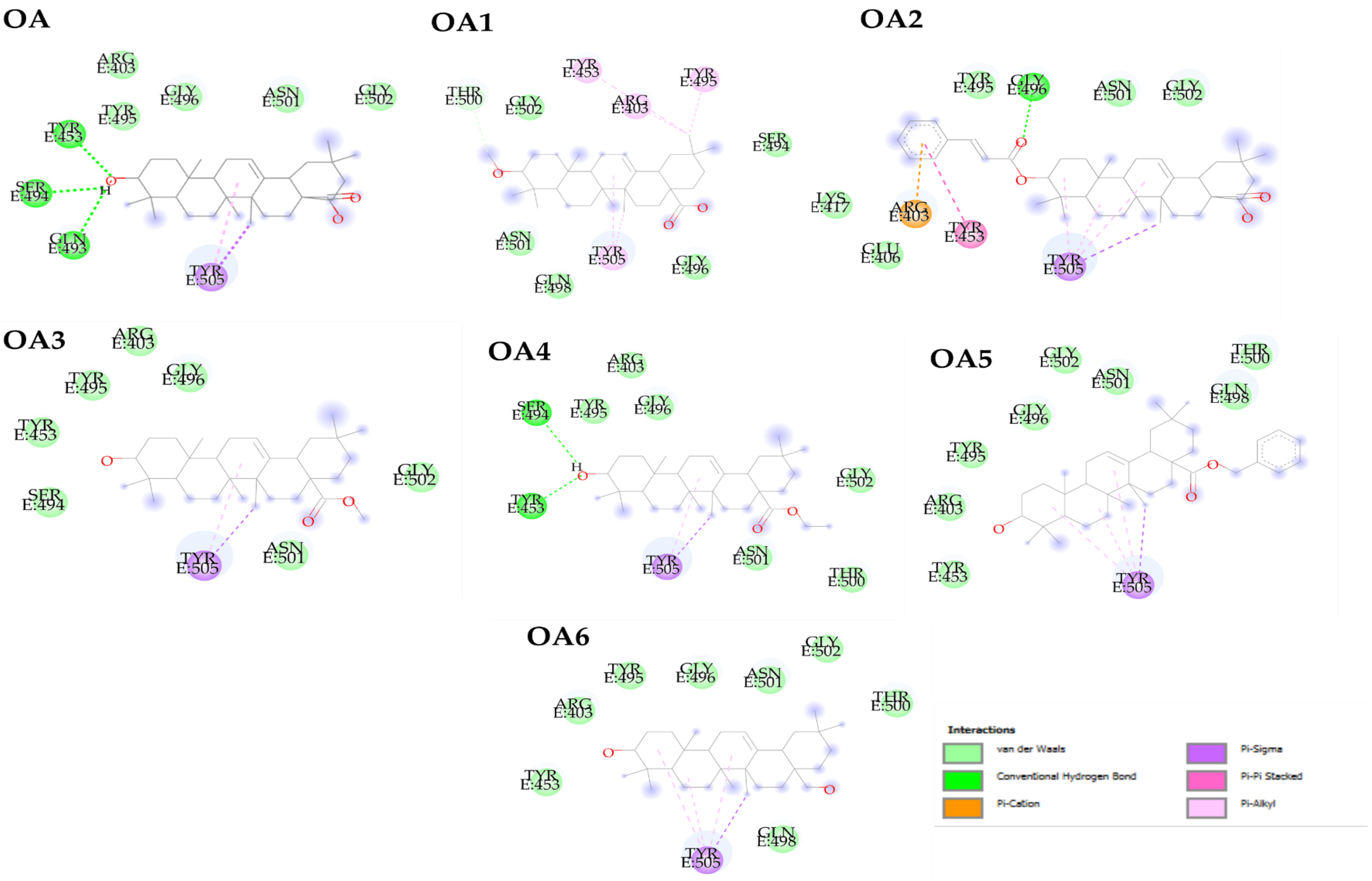

Figure S1. 2D interaction diagram for all triterpene derivatives. All diagrams were constructed with Discovery studio 2021. Interaction type colors labels are dis-played.

Figure S1. Continuation

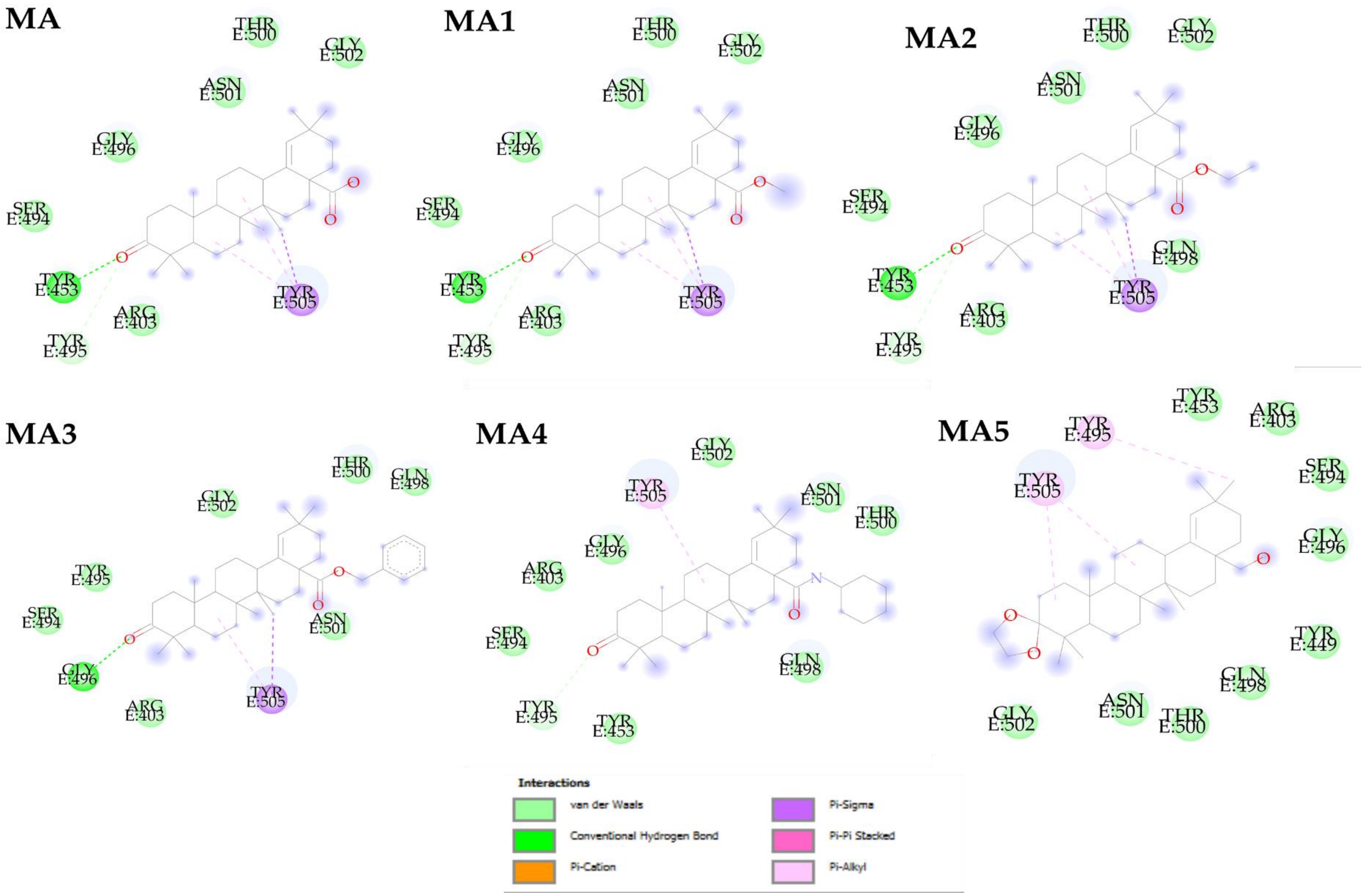

Figure S1. Continuation

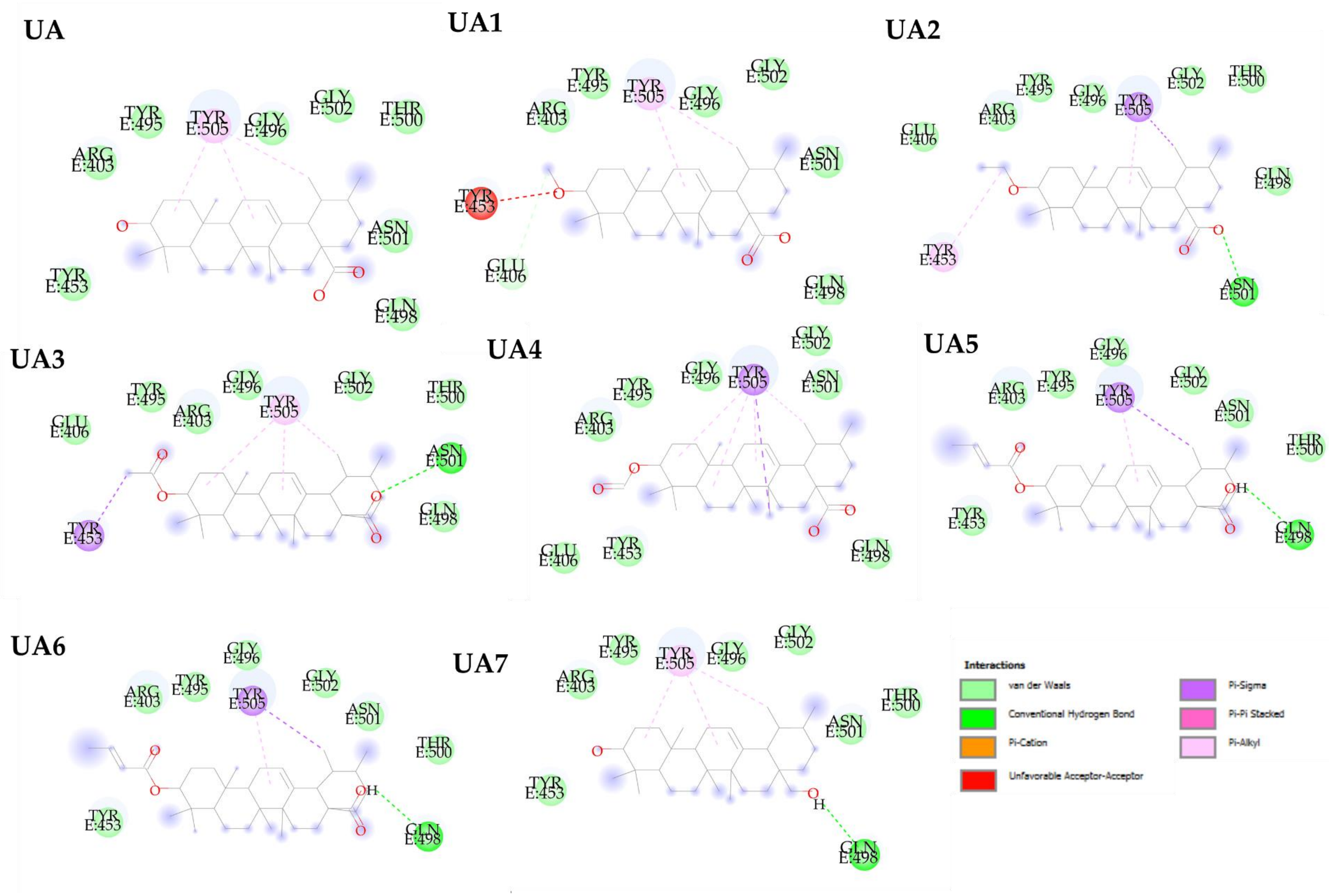

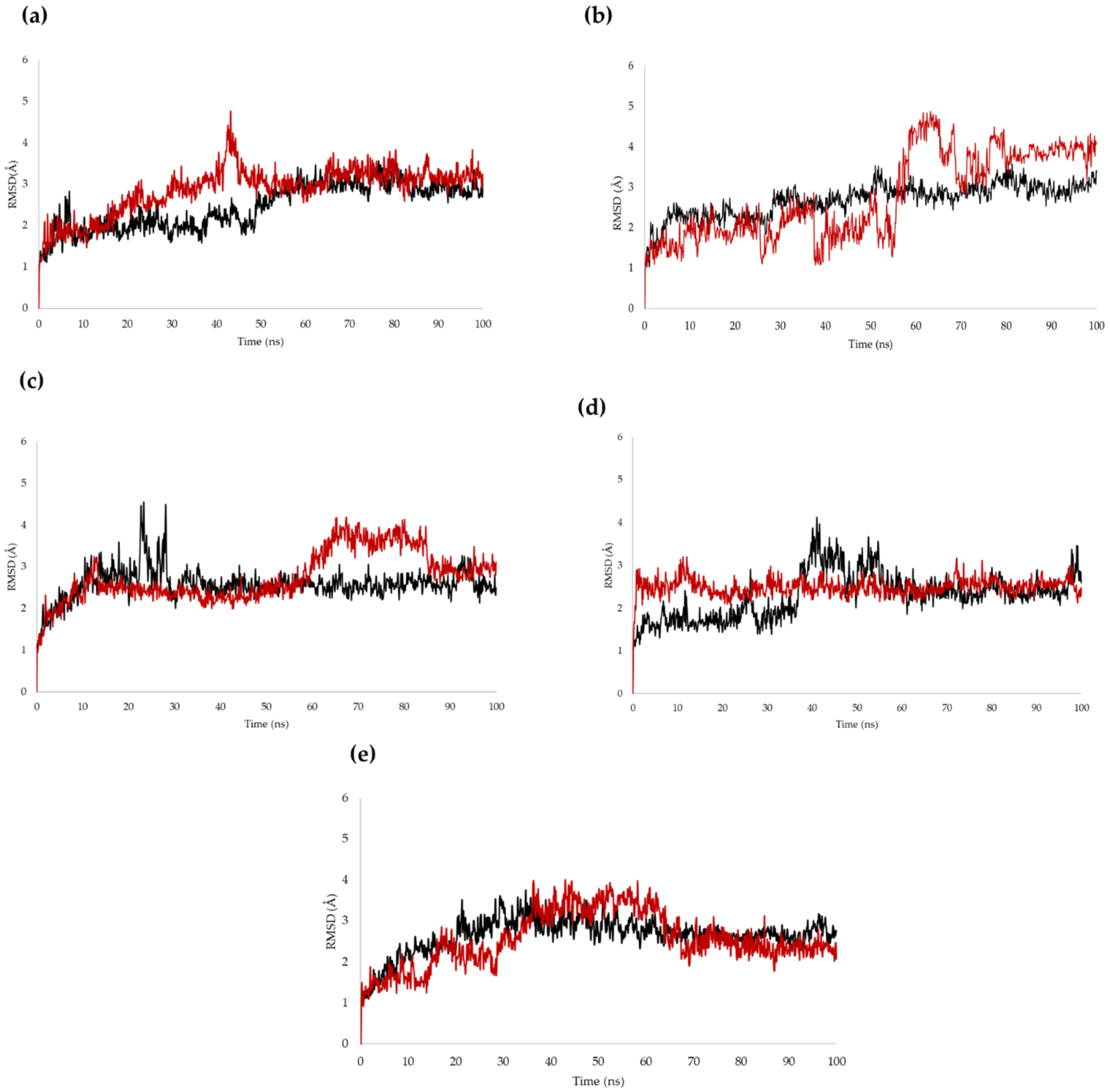

Figure S2. RMSD protein backbone values: (a) RBD domain, (b) GA-RBD complex, (c) **OA5**-RBD complex, (d) **MA4**-RBD complex, E) **UA2**-RBD complex. Black representation corresponds to replica 1, red to replica 2.

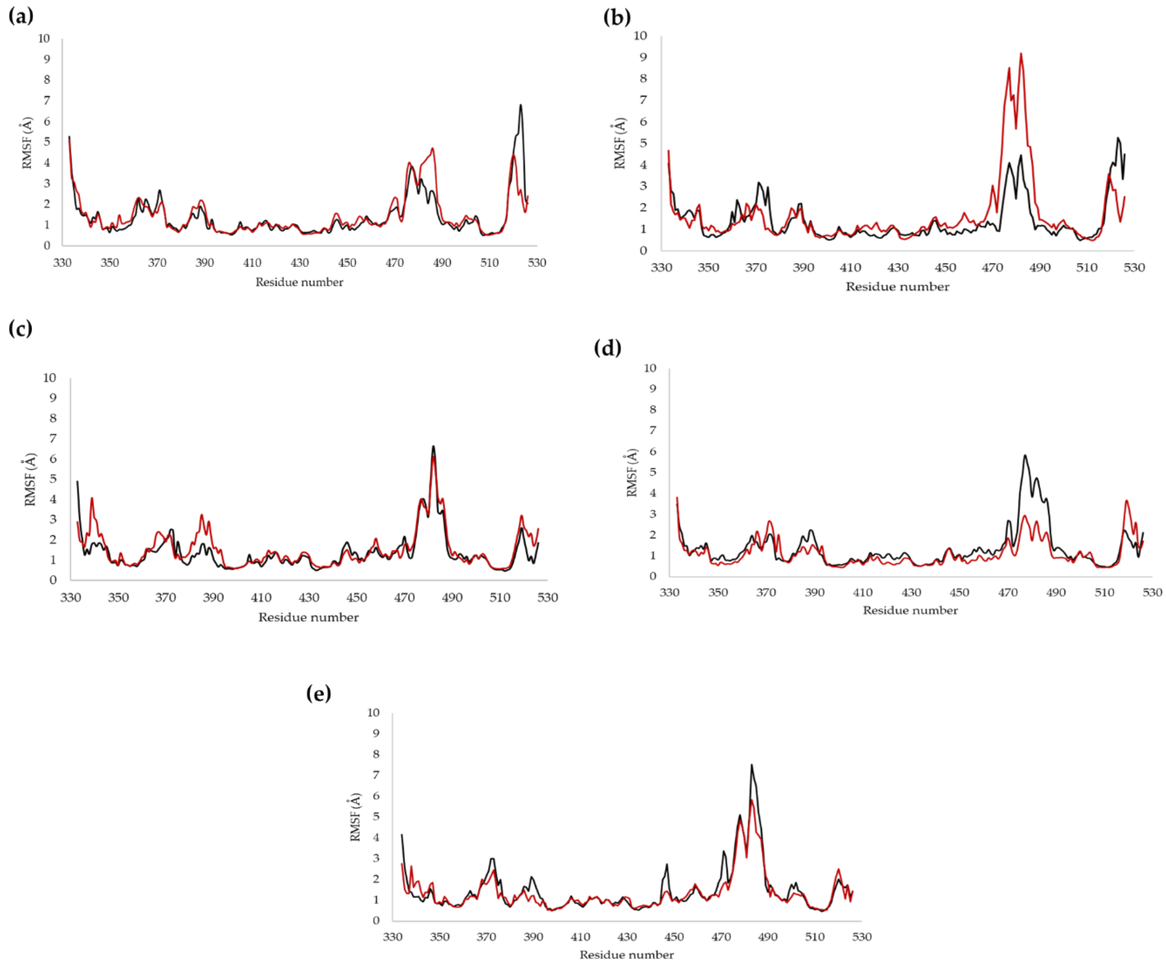

Figure S3. RMSF CA values: (a) RBD domain, (b) GA-RBD complex, (c) **OA5**-RBD complex, (d) **MA4**-RBD complex, (e) **UA2**-RBD complex. Black representation corresponds to replica 1, red to replica 2.

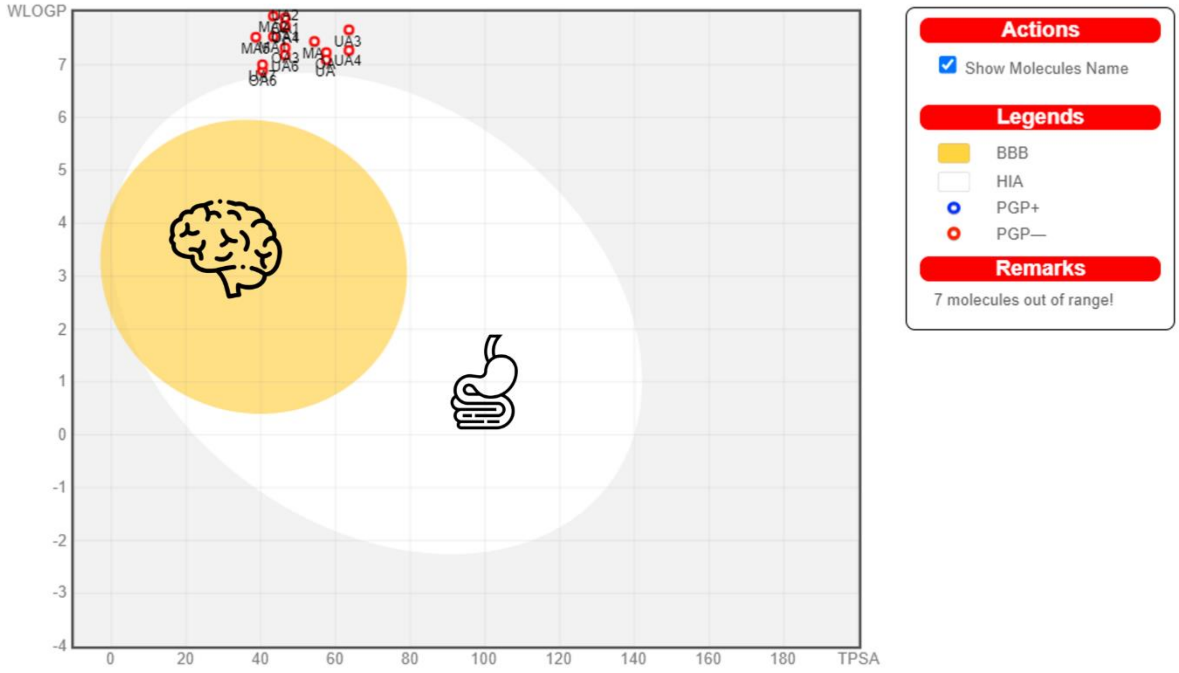

Figure S4. Egan's boiled egg diagram for all the studied compounds and the reference molecule. Neither molecule is a substrate of the P-gp.

Table S1. Secondary structure percentage from the final frame in the 100 ns molecular dynamics simulation.

	RBD	**OA5**	**MA5**	**UA2**	GA
**α-helix**	11.3	12.6	10.8	13.1	10.6
**B sheet**	27.1	24.2	28.4	23.9	27.1

Table S2. RMSF values, in Å, for the region T470-F490 for both replicas.

	RBD		**OA5**		**MA5**		**UA2**		GA	
**Residue number**	**Replica 1**	**Replica 2**	**Replica 1**	**Replica 2**	**Replica 1**	**Replica 2**	**Replica 1**	**Replica 2**	**Replica 1**	**Replica 2**
**470**	1.81	2.36	2.18	1.77	2.69	1.86	3.38	1.79	1.28	3.06
**471**	1.87	2.26	1.54	1.83	2.61	1.54	3.08	1.88	1.15	2.49
**472**	1.44	1.38	1.13	1.48	1.48	1.13	1.84	1.48	0.95	1.80
**473**	1.46	1.19	1.13	1.62	1.72	1.01	2.05	1.89	0.91	3.27
**474**	1.99	1.87	1.68	1.93	2.32	1.36	2.37	2.35	1.85	4.62
**475**	2.49	3.42	2.44	2.70	4.02	2.03	3.75	3.22	2.73	6.72
**476**	3.29	4.03	3.42	3.68	4.90	2.67	4.59	4.28	3.52	7.57
**477**	3.84	3.82	3.98	4.05	5.83	2.95	5.11	4.87	4.10	8.54
**478**	3.67	3.59	4.03	3.67	5.47	2.65	4.57	4.56	3.83	7.00
**479**	3.18	2.99	3.64	3.57	4.94	2.37	4.02	4.02	3.41	7.27
**480**	2.52	2.97	3.19	3.30	3.84	1.73	3.23	3.04	2.43	5.69
**481**	3.23	3.87	5.12	4.85	4.50	2.35	5.09	4.95	3.94	7.62
**482**	2.94	4.02	6.64	6.13	4.75	2.67	7.53	5.82	4.47	9.22
**483**	2.76	4.15	5.65	5.33	4.30	2.06	6.88	5.45	3.33	8.35
**484**	2.05	4.31	3.40	4.11	3.70	1.71	6.49	4.27	2.99	6.67
**485**	2.63	4.42	3.30	3.80	3.57	1.94	5.26	4.13	2.84	4.90
**486**	2.64	4.72	3.46	4.04	3.75	2.14	4.41	3.93	1.77	4.84
**487**	2.30	3.86	2.47	3.12	3.02	1.72	3.21	2.94	1.54	3.90
**488**	1.61	2.16	1.51	2.03	1.66	1.08	1.72	2.16	0.99	2.25
**489**	1.25	1.90	1.17	1.59	1.26	0.90	1.39	1.90	1.18	2.03
**490**	1.04	1.32	1.11	1.23	1.38	0.91	1.75	1.16	1.16	1.46

Table S3. Structural and physicochemical properties for all the derivatives and the reference molecule.

Compound	M. Wt g/mol	TPSA Å²	Log P o/w		LogS (ESOL)	HBA	HBD	Rotables bonds	Druglikeness*					Bioavailability score
			WLOGP	MLOGP					Lipinski	Ghose	Veber	Egan	Mugue	
GA	833.01	279.68	-0.20	-0.67	-6.05	16	12	7	3	3	1	1	4	0.17
RS	602.58	213.36	2.21	0.18	-4.12	12	4	14	2	3	2	1	3	0.17
UM	477.41	80.00	4.87	3.59	-5.45	4	1	8	0	0	0	0	0	0.55
OA	456.70	57.53	7.23	5.82	-7.32	3	2	1	1	3	0	1	1	0.85
OA1	470.73	46.53	7.89	6.01	-7.68	3	2	2	1	3	0	1	1	0.85
OA2	586.84	63.60	9.39	6.98	-9.65	4	1	5	2	4	0	1	1	0.85
OA3	470.73	46.53	7.32	6.01	-7.55	3	1	2	1	3	0	1	1	0.55
OA4	484.75	46.53	7.71	6.20	-7.80	3	1	3	1	4	0	1	1	0.55
OA5	546.02	46.53	8.74	6.83	-8.94	3	1	4	2	4	0	1	1	0.17
OA6	428.69	40.46	6.89	5.80	-6.77	2	2	0	1	3	0	1	1	0.55
MA	454.68	54.37	7.44	5.73	-7.27	3	1	1	1	3	0	1	1	0.85
MA1	468.71	43.37	7.53	5.92	-7.49	3	0	2	1	3	0	1	1	0.55
MA2	482.74	43.37	7.92	6.10	-7.74	3	0	3	1	4	0	1	1	0.55
MA3	544.81	43.37	8.95	6.75	-8.89	3	0	4	2	4	0	1	1	0.17
MA4	535.84	46.17	8.81	6.43	-8.62	2	1	3	2	4	0	1	1	0.17
MA5	535.84	46.17	7.52	5.90	-8.62	2	1	3	2	4	0	1	1	0.17
UA	456.70	57.53	7.09	5.82	-7.23	3	2	1	1	3	0	1	1	0.85
UA1	470..73	46.53	7.74	6.01	-7.59	3	1	2	1	3	0	1	1	0.85
UA2	484.75	46.53	8.13	6.20	-7.64	3	1	3	1	4	0	1	1	0.85
UA3	498.74	63.60	7.66	6.06	-7.72	4	1	3	1	4	0	1	1	0.85
UA4	484.71	63.60	7.27	5.88	-7.64	4	1	3	1	4	0	1	1	0.85
UA5	524.77	63.60	8.33	6.34	-8.34	4	1	4	1	4	0	1	1	0.85
UA6	470.73	46.53	7.18	6.01	-7.46	3	1	2	1	3	0	1	1	0.55
UA7	442.72	40.46	7.00	6.00	-7.19	2	2	1	1	3	0	1	1	0.55

* Number of rule violations.

Table S4. Pharmacokinetic and medicinal chemistry properties for all the studied compounds.

Compound	Pharmacokinetics								Medicinal chemistry		
	GI absorption	BBB permeant	P-gp substrate	CY1A2 inhibitor	CYP2C19 inhibitor	CYP2C9 inhibitor	CYP2D6 inhibitor	CYP3A4 inhibitor	PAINS	Brenk	Leadlikeness
GA	Low	No	Yes	No	No	No	No	No	0	2	1
RS	Low	No	Yes	No	No	No	No	Yes	0	1	2
UM	High	No	No	No	Yes	Yes	Yes	Yes	1	0	3
OA	Low	No	No	No	No	No	No	No	0	1	2
OA1	Low	No	No	No	No	No	No	No	0	1	2
OA2	Low	No	No	No	No	No	No	No	0	2	2
OA3	Low	No	No	No	No	No	No	No	0	1	2
OA4	Low	No	No	No	No	No	No	No	0	1	2
OA5	Low	No	No	No	No	No	No	No	0	1	2
OA6	Low	No	No	No	No	No	No	No	0	1	2
MA	Low	No	No	No	No	Yes	No	No	0	1	2
MA1	Low	No	No	No	No	No	No	No	0	1	2
MA2	Low	No	No	No	No	No	No	No	0	1	2
MA3	Low	No	No	No	No	No	No	No	0	1	2
MA4	Low	No	No	No	No	No	No	No	0	1	2
MA5	Low	No	No	No	No	No	No	No	0	1	2
UA	Low	No	No	No	No	No	No	No	0	1	2
UA1	Low	No	No	No	No	No	No	No	0	1	2
UA2	Low	No	No	No	No	No	No	No	0	1	2
UA3	Low	No	No	No	No	No	No	No	0	1	2
UA4	Low	No	No	No	No	Yes	No	No	0	2	2
UA5	Low	No	No	No	No	Yes	No	No	0	2	2
UA6	Low	No	No	No	No	No	No	No	0	1	2
UA7	Low	No	No	No	No	No	No	No	0	1	2

Table S5. SMILES of the studied compounds and the reference compound (GA).

Compound key name	SMILES
GA	C1C[C@](C)(C(O)O)C[C@H]([C@@]12C)C3[C@](C)(CC2)[C@]4(C)[C@H](C(C3)O)[C@]5(C)[C@@H](CC4)C(C)(C)[C@@H](CC5)O[C@H]6[C@@H]([C@H](O)[C@H](O)[C@@H](O6)C(O)O)O[C@H]7[C@H](O)[C@H](O)[C@H](O)[C@@H](O7)C(O)O
OA	C1C[C@H](O)C(C)(C)[C@H](CC2)[C@@]1(C)[C@H]([C@@]23C)CC=C4[C@@]3(C)CC[C@]5(C(=O)O)[C@H]4CC(C)(C)CC5
**OA1**	C1C[C@H](OC)C(C)(C)[C@H](CC2)[C@@]1(C)[C@H]([C@@]23C)CC=C4[C@@]3(C)CC[C@]5(C(=O)O)[C@H]4CC(C)(C)CC5
**OA2**	c1ccccc1\C=C\C(=O)O[C@@H](CC2)C(C)(C)[C@H](CC3)[C@@]2(C)[C@H]([C@@]34C)CC=C5[C@@]4(C)CC[C@]6(C(=O)O)[C@H]5CC(C)(C)CC6
**OA3**	C1C[C@H](O)C(C)(C)[C@H](CC2)[C@@]1(C)[C@H]([C@@]23C)CC=C4[C@@]3(C)CC[C@]5(C(=O)OC)[C@H]4CC(C)(C)CC5
**OA4**	C1C[C@H](O)C(C)(C)[C@H](CC2)[C@@]1(C)[C@H]([C@@]23C)CC=C4[C@@]3(C)CC[C@]5(C(=O)OCC)[C@H]4CC(C)(C)CC5
**OA5**	C1C[C@H](O)C(C)(C)[C@H](CC2)[C@@]1(C)[C@H]([C@@]23C)CC=C4[C@@]3(C)CC[C@@]5([C@H]4CC(C)(C)CC5)C(=O)OCc6ccccc6
**OA6**	C1C[C@H](O)C(C)(C)[C@H](CC2)[C@@]1(C)[C@H]([C@@]23C)CC=C4[C@@]3(C)CC[C@]5(O)[C@H]4CC(C)(C)CC5
MA	C1CC(=O)C(C)(C)[C@H](CC2)[C@@]1(C)[C@H]([C@@]23C)CC[C@H]4[C@@]3(C)CC[C@]5(C(=O)O)C4=CC(C)(C)CC5
**MA1**	C1CC(=O)C(C)(C)[C@H](CC2)[C@@]1(C)[C@H]([C@@]23C)CC[C@H]4[C@@]3(C)CC[C@]5(C(=O)OC)C4=CC(C)(C)CC5
**MA2**	C1CC(=O)C(C)(C)[C@H](CC2)[C@@]1(C)[C@H]([C@@]23C)CC[C@H]4[C@@]3(C)CC[C@]5(C(=O)OCC)C4=CC(C)(C)CC5
**MA3**	C1CC(=O)C(C)(C)[C@H](CC2)[C@@]1(C)[C@H]([C@@]23C)CC[C@H]4[C@@]3(C)CC[C@@]5(C4=CC(C)(C)CC5)C(=O)OCc6ccccc6
**MA4**	C1CC(=O)C(C)(C)[C@H](CC2)[C@@]1(C)[C@H]([C@@]23C)CC[C@H]4[C@@]3(C)CC[C@@]5(C4=CC(C)(C)CC5)C(=O)NC6CCCCC6
**MA5**	C1C(C)(C)CC[C@@](CC2)(CO)C=1[C@@H](CC3)[C@]2(C)[C@](C)(CC4)[C@H]3[C@@](C)(CC5)[C@@H]4C(C)(C)C56OCCO6
UA	C1C[C@H](O)C(C)(C)[C@H](CC2)[C@@]1(C)[C@H]([C@@]23C)CC=C4[C@@]3(C)CC[C@]5(C(=O)O)[C@H]4[C@@H](C)[C@H](C)CC5
**UA1**	C1C[C@H](OC)C(C)(C)[C@H](CC2)[C@@]1(C)[C@H]([C@@]23C)CC=C4[C@@]3(C)CC[C@]5(C(=O)O)[C@H]4[C@@H](C)[C@H](C)CC5
**UA2**	CCO[C@@H](CC1)C(C)(C)[C@H](CC2)[C@@]1(C)[C@H]([C@@]23C)CC=C4[C@@]3(C)CC[C@]5(C(=O)O)[C@H]4[C@@H](C)[C@H](C)CC5
**UA3**	CC(=O)O[C@@H](CC1)C(C)(C)[C@H](CC2)[C@@]1(C)[C@H]([C@@]23C)CC=C4[C@@]3(C)CC[C@]5(C(=O)O)[C@H]4[C@@H](C)[C@H](C)CC5
**UA4**	O=CO[C@@H](CC1)C(C)(C)[C@H](CC2)[C@@]1(C)[C@H]([C@@]23C)CC=C4[C@@]3(C)CC[C@]5(C(=O)O)[C@H]4[C@@H](C)[C@H](C)CC5
**UA5**	C\C=C\C(=O)O[C@@H](CC1)C(C)(C)[C@H](CC2)[C@@]1(C)[C@H]([C@@]23C)CC=C4[C@@]3(C)CC[C@]5(C(=O)O)[C@H]4[C@@H](C)[C@H](C)CC5
**UA6**	C1C[C@H](O)C(C)(C)[C@H](CC2)[C@@]1(C)[C@H]([C@@]23C)CC=C4[C@@]3(C)CC[C@]5(C(=O)OC)[C@H]4[C@@H](C)[C@H](C)CC5
**UA7**	C1C[C@H](O)C(C)(C)[C@H](CC2)[C@@]1(C)[C@H]([C@@]23C)CC=C4[C@@]3(C)CC[C@]5(CO)[C@H]4[C@@H](C)[C@H](C)CC5

Table S6. Molecular docking scores of the top selected compounds with N=10 from 10 independently generated random seeds. The most favorable energy binding is shown for each seed.

GA		OA5		MA4		UA2	
**Seed**	**Docking score (kcal/mol)**	**Seed**	**Docking score (kcal/mol)**	**Seed**	**Docking score (kcal/mol)**	**Seed**	**Docking score (kcal/mol)**
26347	-7.6	68517	-7.3	15185	-7.4	22726	-7.3
22498	-7.6	32508	-7.3	13801	-7.4	19130	-7.2
80789	-7.6	14357	-7.3	77280	-7.4	59177	-7.2
10812	-7.6	39207	-7.3	13010	-7.4	56546	-7.2
87253	-7.6	69976	-7.3	72965	-7.4	64974	-7.2
42615	-7.6	13174	-7.3	71367	-7.4	13334	-7.2
34894	-7.6	75915	-7.3	12385	-7.4	70594	-7.2
61701	-7.6	74686	-7.3	80301	-7.4	93085	-7.2
91542	-7.6	93755	-7.3	11239	-7.4	18044	-7.2
70874	-7.2	85230	-7.3	58405	-7.4	47198	-7.2
